# Mechanism of herpesvirus protein kinase UL13 in immune escape and viral replication

**DOI:** 10.3389/fimmu.2022.1088690

**Published:** 2022-11-30

**Authors:** Lin Zhou, Anchun Cheng, Mingshu Wang, Ying Wu, Qiao Yang, Bin Tian, Xumin Ou, Di Sun, Shaqiu Zhang, Sai Mao, Xin-Xin Zhao, Juan Huang, Qun Gao, Dekang Zhu, Renyong Jia, Mafeng Liu, Shun Chen

**Affiliations:** ^1^ Institute of Preventive Veterinary Medicine, Sichuan Agricultural University, Chengdu, Sichuan, China; ^2^ Key Laboratory of Animal Disease and Human Health of Sichuan Province, Sichuan Agricultural University, Chengdu, Sichuan, China; ^3^ Avian Disease Research Center, College of Veterinary Medicine, Sichuan Agricultural University, Chengdu, Sichuan, China

**Keywords:** UL13, serine/threonine protein kinase, immune escape, viral replication, cGAS-STING, NF-κB

## Abstract

Upon infection, the herpes viruses create a cellular environment suitable for survival, but innate immunity plays a vital role in cellular resistance to viral infection. The UL13 protein of herpesviruses is conserved among all herpesviruses and is a serine/threonine protein kinase, which plays a vital role in escaping innate immunity and promoting viral replication. On the one hand, it can target various immune signaling pathways *in vivo*, such as the cGAS-STING pathway and the NF-κB pathway. On the other hand, it phosphorylates regulatory many cellular and viral proteins for promoting the lytic cycle. This paper reviews the research progress of the conserved herpesvirus protein kinase UL13 in immune escape and viral replication to provide a basis for elucidating the pathogenic mechanism of herpesviruses, as well as providing insights into the potential means of immune escape and viral replication of other herpesviruses that have not yet resolved the function of it.

## Introduction

Herpes virus is a virus of double-stranded DNA that can be divided into three subfamilies: α-, β-, and γ-herpesvirus. For instance, Herpes simplex virus type 1/2 (HSV1/2), Varicella-zoster virus (VZV), Pseudorabies virus (PRV), Epstein-Barr virus (EBV), Human cytomegalovirus (HCMV), Kaposi’s sarcoma-associated herpesvirus (KSHV), Murine gamma-herpesvirus 68(MHV-68), Marek’s disease virus (MDV) and Duck plague virus (DPV) (1–3). Herpes virus infections severely impact the health of humans and animals. The host’s innate immune system is the first line of defense against invading pathogens, it relies on the mutual recognition of various pathogen recognition receptors (PRR) and pathogen-associated molecular patterns (PAMP) on the surface of the pathogenic organism. The interaction between PRR and PAMP on the surface of pathogenic organisms induces the production of Type I interferon (IFN-I) and other antiviral factors, promoting cellular antiviral immunity and activating the corresponding immune system (4). cyclic GMP-AMP synthase (cGAS) is a nucleotidyltransferase, as a member of the PRR family, which is activated by binding viral double-stranded DNA to induce the production of IFN-ß (5, 6). The nuclear factor kappa-B (NF-κB) regulates the production of inflammatory and immune responses to protect the host from pathogens (7, 8). Similarly, the JAK-STAT signaling pathway, the PKR-eIF2α signaling pathway, the Sterile alpha motif and HD domain-containing protein 1 (SAMHD1), and the CD8+ T cell play a critical role in antiviral response.

The HSV pUL13 and its homologs (e.g., EBV pBGLF4, HCMV pUL97, KSHV pORF36, MHV-68 pORF36, and VZV pORF47) are serine/threonine protein kinase belonging to the conserved herpesvirus protein kinase family (CHPK), which is a tegument protein of herpes virions (9–11). Their catalytic core consists of 12 conserved subdomains (12–15) (**Table 1**), which can catalyze the transfer of the γ-phosphate of a nucleoside triphosphate to amino acid residues of protein substrates to affect their function. CHPK from different herpesvirus subfamilies has considerable amino acid variation, and there is no consensus phosphorylation sequence for all CHPKs (16–18). Moreover, the herpesvirus protein kinases have very low homology with known cell kinases.

**Table 1:** The Protein kinase catalytic subdomain.

Conserved subdomain	Conserved amino acid	Function
I	Gly-X-Gly-X-X-GLy-X-Va1	Anchor the ATP
II	Lys	proton transfer
III	Glu	Stabilizing the interaction between the functional subdomain II Lys and the α and β phosphate groups of ATP
IV	/	/
V	/	/
VIA	/	Supporting action
VIB	Asp,Asn	Asn interacts with Asp to stabilize ATP and bind Mg2+ to form a salt bridge
VII	Asp, Gly	Orientation of ATP
VIII	Ala,Pro,Glu	Identification of substrate
IX	Asp, Gly	Hydrogen-bonded with Arg of subdomain VIB to stabilize the catalytic ring.
X	/	/
XI	Arg	Stabilization

There are no conserved amino acids among the subdomains IV, V, VIA and X. "/" indicates that there is no Conserved amino acid site in "conserved amino acid" and that the Function is not clear in "function".

With the continuous discovery of UL13 protein kinase substrates (**Table 2**), pUL13 has been shown to play an important role in the physiological activity of the herpes virus. For example, VZV pORF47 and KSHV pORF36 are essential for virus proliferation in T and B cells (19–21); PRV pUL13 affects IFN- β by inhibiting zinc finger CCHC-type containing protein 3 (ZCCHC3) expression (22); EBV pBGLF4 is a regulator of the EBV immune genes BCRF1 and BPLF1 (23). Moreover, HSV-2 pUL13 Ser18 was significantly crucial for the HSV-2 capacity of replication and cell-to-cell spread in U2OS cells (24); the deletion of pUL13 reduced the size and number of Viral plaques of DPV (25); CHPK of β and γ herpes viruses promotes DNA virus replication by mimicking cyclin-dependent kinases1/2 (CDK1/2) phosphorylation of cyclin (26, 27). These suggest that pUL13 plays an essential role in immune escape and viral replication of herpes viruses.

**Table 2:** The substrates of herpes virus UL13 protein kinase.

Protein	Substrates	
	Cellular proteins	Viral proteins
**UL13**	**STING**	**BRMF1/4**
	**IRF-3**	**EBNA-LP**
	**PRDX1**	**PP65**
	**UXT**	**U69**
	**SAMDH1**	**UL41**
	**PKR**	**UL44**
	**Rb**	**UL49**
	**CKIIβ**	**ICP22**
	**EF-1δ**	**ICP0**
	**H2AX**	**gE/gI**
	**H2B**	**US3**
	**LaminA/C**	**IE62/IE63**
	**RNA pol II**	**VP13/14**
	**AKT**	**K-bZIP**
	**JNK**	**BZLF1**
	**p60**	**EBNA2**
	**HADC1/2**	**EA-D**
	**Tip60**	**ORF9**
	**LANA**	**ORF36**

UL13: represents the conserved herpesvirus protein kinase of all herpesviruses, such as EBV BGLF4 and KSHV ORF36.

pUL13, dependent or independent of its kinase activity, regulates Cellular and Viral proteins that affect innate immunity and the cell cycle to promote viral replication.

## The role of pUL13 in viral evasion of innate immunity

### Inhibition of the cGAS/STING pathway

**Figure 1:**
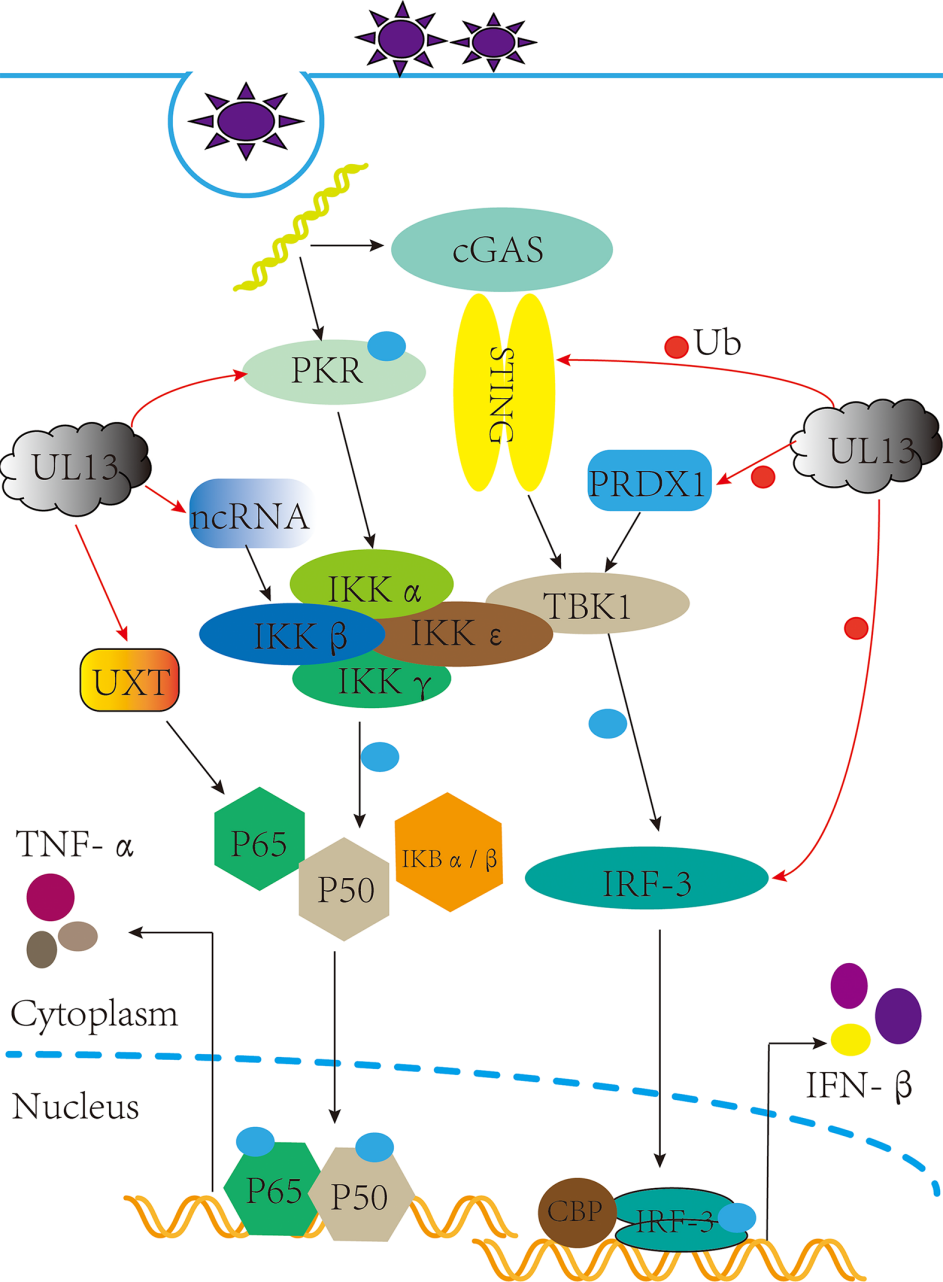
pUL13 inhibits the cGAS-STING signaling pathway and the NF-κB signaling pathway. pUL13 can inhibit the dimerization, nuclear translocation, CBP binding, and binding to IFN-β promoter elements of interferon regulatory factor 3 (IRF3); meanwhile, it can ubiquitinally degrade stimulator of interferon genes (STING), IRF-3, and peroxidase 1 (PRDX1) to inhibit the production of IFN-β. pUL13 indirectly inhibits the NF-κB pathway by regulating ubiquitously expressed transcript (UXT), Protein kinase R (PKR), and B2 SINE ncRNA.

The type I interferon pathway is a significant component of innate immunity and plays an essential role in the control and clearance of pathogens. Upon infection, inhibition of interferon regulatory factor 3 (IRF3) by viral infection is a critical link for the termination of the type I interferon pathway. Here, IRF3 is an essential target for pUL13 action during herpes virus infection because many studies have shown that they can phosphorylate IRF3 and inhibit IRF3 dimerization, binding to the positive regulatory domains III-I (PRDIII-I), and interaction with the CREB-binding protein and P300 protein (CBP/P300) (28–32). Meanwhile, Lin Lv et al. showed that PRV UL13 relies on its kinase active sites of Lys49 and Lys387 to target IRF3 and promote its ubiquitination for degradation by the proteasome (33) (**Figure 1**).

In addition, pUL13 promotes the ubiquitinated degradation of immunomodulatory proteins as a necessary action affecting innate immunity. For example, PRV pUL13 recruits the E3 ligase RING-finger protein 5 (RNF5) to degrade the stimulator of interferon genes protein (STING) indirectly and also participates in ubiquitination degradation of the host protein peroxidase 1 (PRDX1) to inhibit innate immunity (34, 35) (**Figure 1**). Tripartite motif (TRIM) proteins play a critical role in the antiviral host response. Based on E3 ubiquitin ligases RNF5, TRIM29 and TRIM30α are responsible for the ubiquitination degradation of STING protein; TRIM18 recruit protein phosphatase 1A (PPM1A) to dephosphorylate TANK binding kinase 1 (TBK1) to suppress the innate immune response (36, 37). We believe that the TRIM family members (such as TRIM29 and TRIM18) may be essential partners of herpesvirus pUL13 in promoting the ubiquitination degradation of host immune proteins. However, there are no reports about the interaction between TRIM family members and pUL13.

### Inhibition of NF-κB pathway

After virus infection, NF-κB is activated and translocated into the nucleus, which induces an inflammatory and immune response to protect the host from the pathogen (38, 39). As a vital component of the immune system, which can be regulated by the ubiquitously expressed transcript (UXT). In 2012, Chang et al. found that EBV pBGLF4 phosphorylated UXT at the Thr3, weakening interaction with p65 to inhibit NF-κB activity (40) (**Figure 1**). Not only did it reveal the role of the conserved herpesvirus protein kinases in evading immune clearance by NF-κB, but it also revealed its essential for promoting the lytic cycle.

Furthermore, short interspersed elements (SINEs) are non-coding retrotransposons transcribed by RNA polymerase III (RNA Pol III), which activate antiviral NF-κB signaling through a mitochondrial antiviral signaling protein (MAVS)-dependent and independent mechanism pathways (41). However, MHV-68 infection can sustainably induce transcription of SINE ncRNA, which is explained by Xiaonan Dong et al.: Inducing phosphorylation degradation of the RelA/p65 subunit of NF-κB in the pre-MHV-68 infection period to blunt the NF-κB transcription response, it is associated with IKKβ kinase (42). In 2020, Aaron M Schaller et al. reported that CHPKs-mediated chromatin modification changes contribute to activating B2 SINEs during MHV68 infection; hijacking uses B2 SINE RNA signal to activate IKKβ kinase and phosphorylates transcription initiation factor Rta to promote viral replication (43) (**Figure 1**). Much more interesting is that the activated SINE ncRNA can directly interact with RNA pol II to participate in the transcriptional suppression of genes (44, 45). By and large, the B2 SINEs seem to do more harm than good for viral replication. Nevertheless, the herpes virus pUL13 chose it, demonstrating that B2 SINEs have many potential mechanisms to be developed in the life cycle of herpes viruses.

### Inhibition of the JAK/STAT signaling pathway

**Figure 2:**
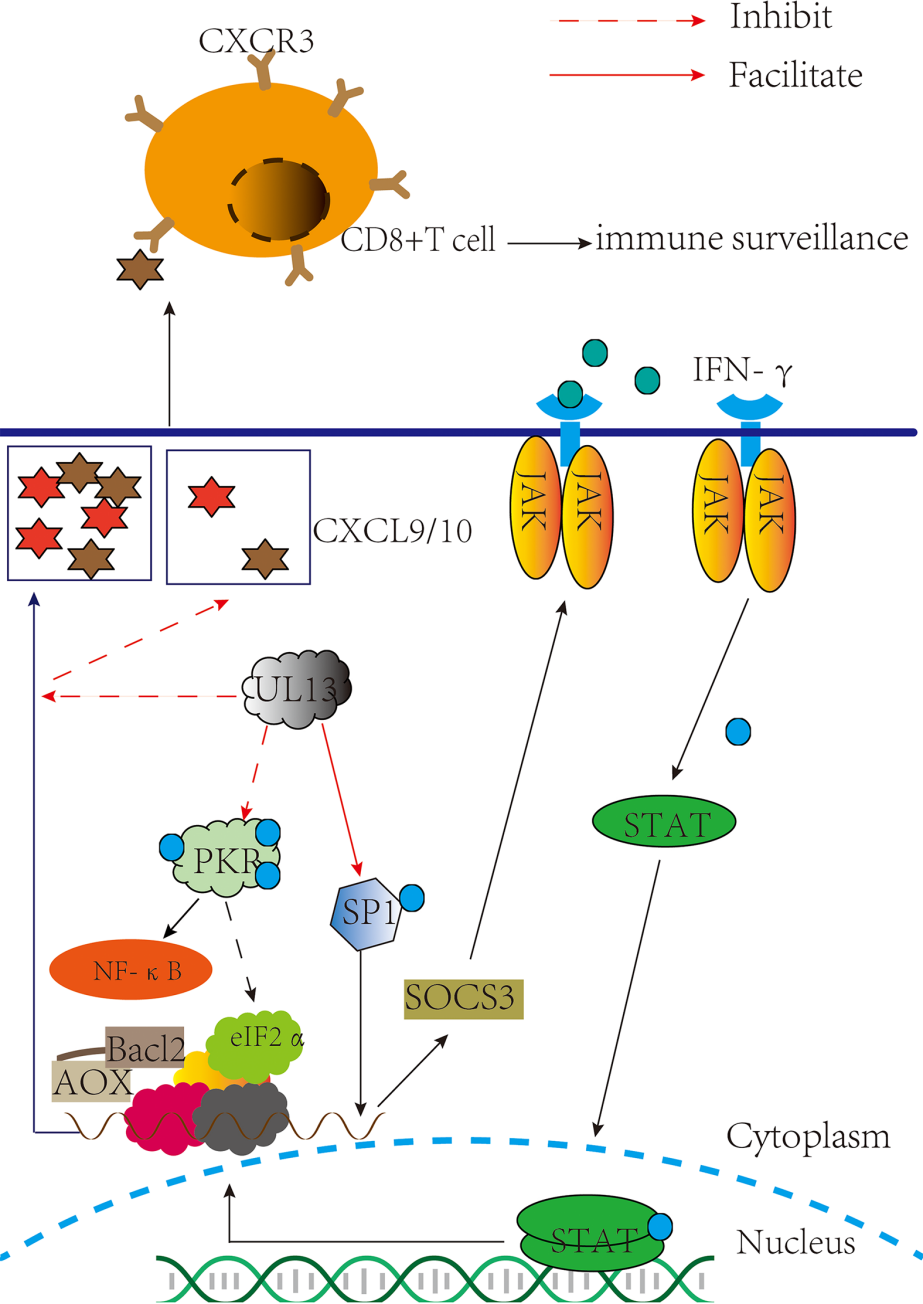
pUL13 inhibits PKR, CD8+ T cells, and the JAK-STAT signaling pathway to evade innate immunity. pUL13 inhibits PKR phosphorylation to evade innate immunity and promotes viral protein translation with eukaryotic initiation factor 2α (eIF2α); pUL13 phosphorylates the associated constitutive transcription factor SP1 (SP1), which induces suppressor of cytokine signaling 3 (SOCS3) expression to inhibit the JAK-STAT signaling pathway; and pUL13 can down-regulate the C-X-C motif chemokine ligand 9 (CXCL9) signaling molecules to prevent cd8+ T cell molecules from clustering at the site of infection.

JAK/STAT acts as an inflammatory signaling pathway for stress and has immunomodulatory effects, receiving multiple cytokine signals from cells, such as IFN-α and IFN-γ (46, 47). In 2017, Yuka Sato et al. reported that pUL13 could phosphorylate the associated constitutive transcription factor SP1 (SP1) to induce suppressor of cytokine signaling 3(SOCS3) production, which regulates the JAK/STAT signaling pathway negatively (48) (**Figure 2**). That is SP1 can combine with GC-rich regions of the SOCS3 promoter to facilitate transcription and translation of SOCS3 (49, 50), and then curb the JAK/STAT signal pathway (51–53). Moreover, the phosphorylation of Sp1 by pUL13 could specifically induce the transcription of the immediate-early and early genes expression of the herpes virus (54–57), which reveals the importance of pUL13 for transcriptional regulation of herpesvirus genes.

SOCS3 plays a significant role in modulating the outcome of infections and autoimmune diseases. And many viruses, such as HSV- 1, EBV, and VZV (58–61), can activate the expression of SOCS3 because of the close relationship between SOCS3 and JAK kinase with STAT signaling factors (62–65). It was suggested that SOCS3 is induced that not only inhibits the antiviral response of the JAK-STAT signal pathway but also maintains immune homeostasis in the body under pathological conditions and physiological conditions (66), such as the expression of SOCS3 inhibits several NF-κB-regulated proapoptotic pathways to protect β-cells from IL- 1 β-mediated apoptosis (67). Perhaps this is more important for the production of SOCS3 induced by pUL13 during herpesvirus infection.

### Effect on PKR-eIF2α-mediated antiviral effects

Protein kinase R (PKR) in host cells exerts antiviral effects by inhibiting viral mRNA translation and inducing apoptosis. Many data indicate PKR promotes NF-κB activation (68–73), promotes mRNA stability of IFN-β (74), and is involved in the tumor suppressor function of p53 protein (75–77). When dsRNA binds to the Conserved double-stranded RNA binding motif (dsRBMs) of PKR, it is activated by autophosphorylation at Thr446 (78). Next, it phosphorylates Ser 51 of eukaryotic initiation factor 2α (eIF2α) and inhibits the translation initiation activity of mRNAs which encode antiviral factors and mediate stress responses (79, 80). In the PKR-eIF2a pathway, PKR inhibition and eIF2a dephosphorylation must be used to achieve massive replication of the virus, so the virus has evolved a variety of strategies in regulating the PKR-eIF2a pathway: controlling dsRNA masking and degradation (81–84), PKR degradation (85), inhibiting PKR dimerization and autophosphorylation (86–89), dephosphorylation of eIF2α (90–92), and PKR desensitization (93, 94). In 2020, Rosamaria Pennisi et al. demonstrated that HSV-1 pUL13 inhibits the phosphorylation of cellular PKR. Although the specific pathway by which pUL13 inhibits PKR phosphorylation cannot be demonstrated (95) (**Figure 2**). These suggest that pUL13 inhibition of PKR can not only evade innate immunity and prevent PKR-mediated apoptosis but also use eIF2α to promote viral mRNA translation.

Especially, PKR is one of four kinases that integrate stress responses. It regulates the protein homeostasis of the cell to maintain the body’s homeostasis; conversely, its abnormal activation can cause severe damage to the body, such as systemic lupus erythematosus (96, 97). Based on these results, whether the molecular mechanism of pUL13 inhibition of PKR can inspire treating diseases associated with abnormal activation of PKR remains to be further studied.

### Effect on CD8+ T cells mediated antiviral effects

Compared with pICP47 and pUS3 recognizing the main histocompatibility complex (MHC I) that are distributed on the cell surface and presentation of antigen peptides to T cells to exert cellular immune clearance regulation, the effect of pUL13 on it is not apparent (98–101). However, HSV- 1 pUL13 triggered viral encephalitis in mice by downregulating CXCL-9 and inhibiting the infiltration of CD8+ T cell molecules at the site of infection (102) (**Figure 2**). The author also points out that the HSV-1 pUL13- mediated immune evasion mechanism might be specific to the CNS. Maybe it associated with CXCL-9/10 and CD8+ T cells inhibiting the reactivation of HSV within nerve cells, further suggesting the role of pUL13 in the latent reactivation of the herpes virus (103–106). Although the molecular mechanisms underlying the downregulation of CXCL-9 by pUL13 are unclear, it is suggested that inhibition of pUL13 has a potential effect in treating encephalitis of the central nervous system caused by HSV-1 infection (107–110).

### Inhibition of SAMHD1

**Figure 3:**
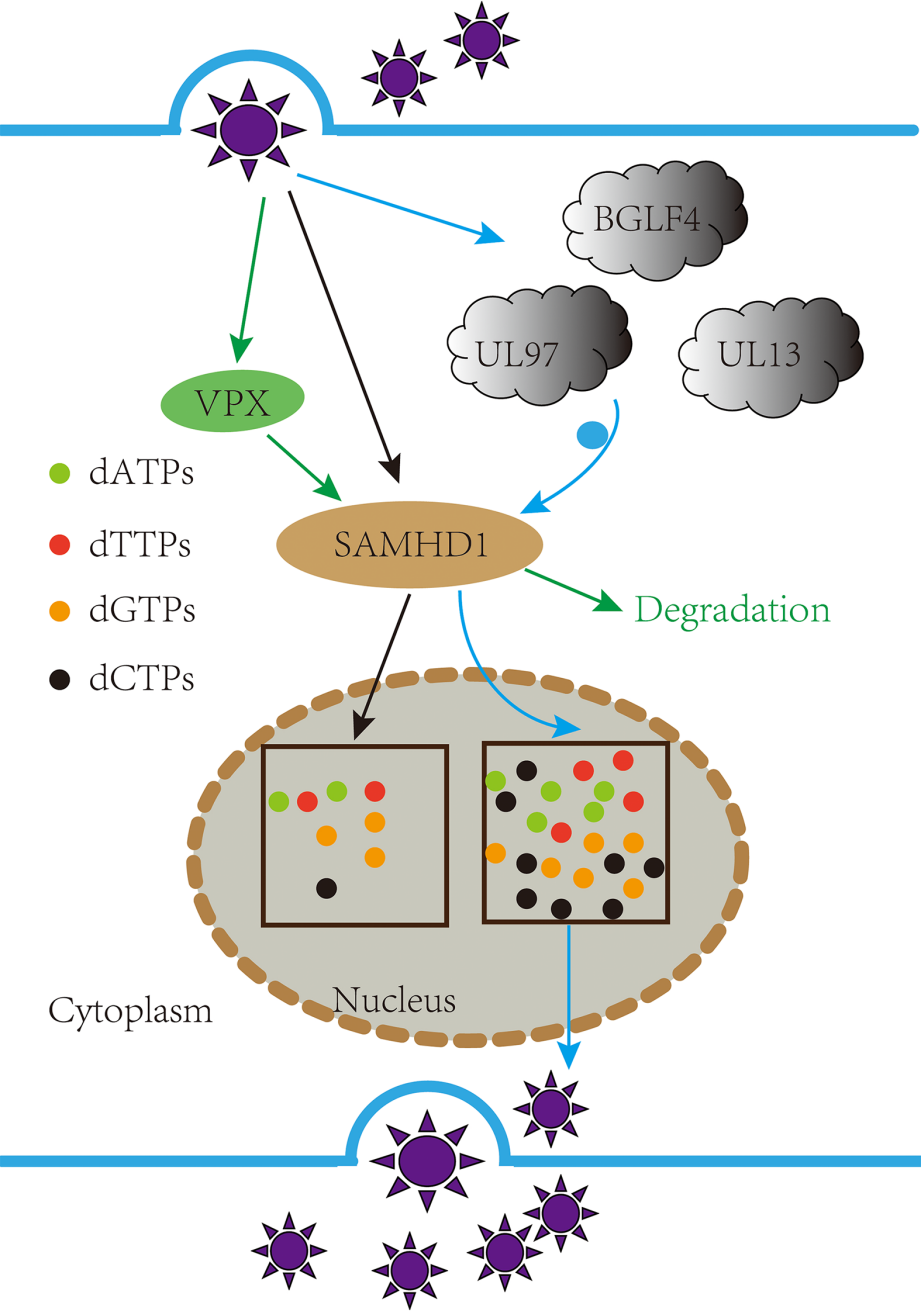
pUL13 phosphorylated the antiviral factor SAMDH1 Thr592 to promote viral replication. Inhibiting the dNTP enzyme activity of the sterile alpha motif and HD domain-containing protein 1 (SAMHD1) from ensuring adequate intracellular levels of dNTPs for viral replication.

SAMHD1 is an antiviral host limiting factor (111–116), and the virus has adopted a variety of strategies to inhibit its dNTP enzyme activity, such as HIV-2 and SIV virus-encoded Vpx proteins, to induce SAMHD1 degradation and promote self-replication (117–120); Ribonucleotide reductase (RNR) (121) and thymidine kinase (TK) (122, 123) encoded by DNA viruses can antagonize SAMHD1’s dNTP enzyme activity, providing the necessary substrate for viral DNA polymerase; The intracellular CyclinA2/CDK1/CDK2 complex regulates phosphorylation of SAMHD1 Thr592 (124), and phosphorylation of Thr592 has been shown to reduce SAMHD1 antiviral activity (125), echoing IFN-I-induced dephosphorylation of SAMHD1 Thr592 (126). It has been also reported that pUL13 of the β and γ herpes virus participates in phosphorylation of SAMHD1 T592, inhibiting the dNTP enzyme activity of SAMHD1 from ensuring adequate intracellular levels of dNTPs for viral replication (127, 128) (**Figure 3**).

SAMHD1 can inhibit the excessive immune and inflammatory response, possibly proving why VZV and KSHV proliferate in lymphocytes requiring pORF47 and pORF36 (129). However, whether and how pUL13 can phosphorylate SMADH1 to coordinate the immune and inflammatory response remains to be studied.

It is revealed here that pUL13 plays an essential role in inhibiting various antiviral factors from escaping innate immunity. Additionally, pUL13 also plays an important role in viral replication, latent infection, and other critical physiological activities.

## The role of pUL13 in promoting viral replication

### pUL13 phosphorylates H2AX to promote viral replication

DNA-damage response (DDR) is a mechanism by which cells protect themselves through DNA damage repair and apoptosis to resist DNA damage induced by various factors (130, 131). Micah A. Luftig has discussed the interrelationship between viruses and DDR, noting that DNA viruses require DDR activation for replication (132). The research shows that the viral infection process acts on the different nodes of the DNA damage response pathway. For example, HSV-1 infections activate ataxia telangiectasia mutated (ATM) kinase activity but inhibit the role of ataxia telangiectasia- and Rad3-related protein (ATR); EBV virus infection activates upstream regulators of the DDR pathway in the DDR pathway-histone acetyltransferase TIP60 (133–135).

**Figure 4:**
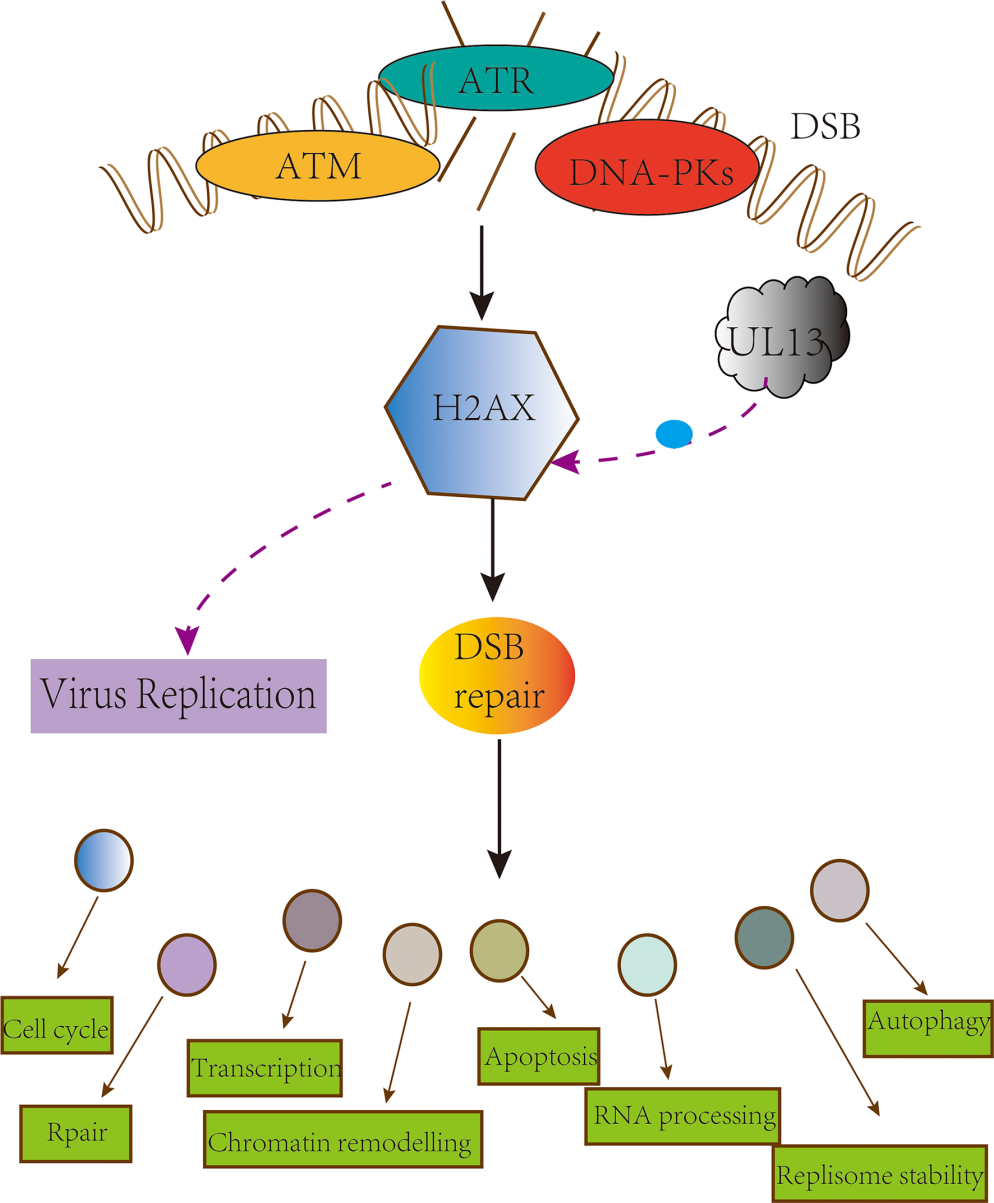
pUL13 phosphorylates H2AX to promote viral replication. The conserved herpesvirus Protein kinase pUL13 regulates DNA damage marker H2A histone family member X (H2AX), and pUL13-mediated H2AX phosphorylation plays a pivotal role in efficient virus replication and progeny production.

H2A histone family member X (H2AX) is a substrate of ATM, ATR, and DNA-dependent protein kinase catalytic subunits in phosphatidylinositol 3-kinase-like protein kinase family (PIKKs) (136–140); it is also a substrate for pUL13 (141, 142). In H2AX knockdown cells, the replication capacity of MHV-68 and KSHV are significantly abating (143, 144), and the date of EBV pBGLF4, PRV pUL13 what suggesting that pUL13 phosphorylate H2AX to activate DDR for viral replication (145, 146). Still, VZV pORF47 cannot phosphorylate H2AX and indicates the difference in the members of the CHPKs (147). An attractive hypothesis is that replication of viral DNA requires or is enhanced by the cellular DNA damage machinery (133, 148–150) (**Figure 4**). Generally, more evidence is needed to support whether pUL13 of the herpes virus plays a vital role in this matter.

### pUL13 phosphorylates EF-1б to promote viral replication

Herpesvirus pUL13 can promote host cell synthesis of proteins, such as the KSHV pORF36 mimicking cellular protein S6 kinase (S6KB1) to promote cell proliferation (151). Similarly, as a substrate of pUL13, the translation extension factor -1б (EF-1б) exists in two forms in the normal state of hypophosphorylation and hyperphosphorylation, involved in the process of mRNA translation into peptide chain extension. EF-1б is mainly present in the hyperphosphorylated form in HSV-1-infected cells. Because HSV-1 pUL13, HCMV pUL97, EBV pBGLF4, and intracellular cycle-dependent kinase cdc2 are involved in EF-1б’s hyperphosphorylation and work together on its Ser 133 (152–154). It shows that UL13 can synthesize its viral protein using EF-1б.

### pUL13 works with SUMO proteins to promote viral replication

**Figure 5:**
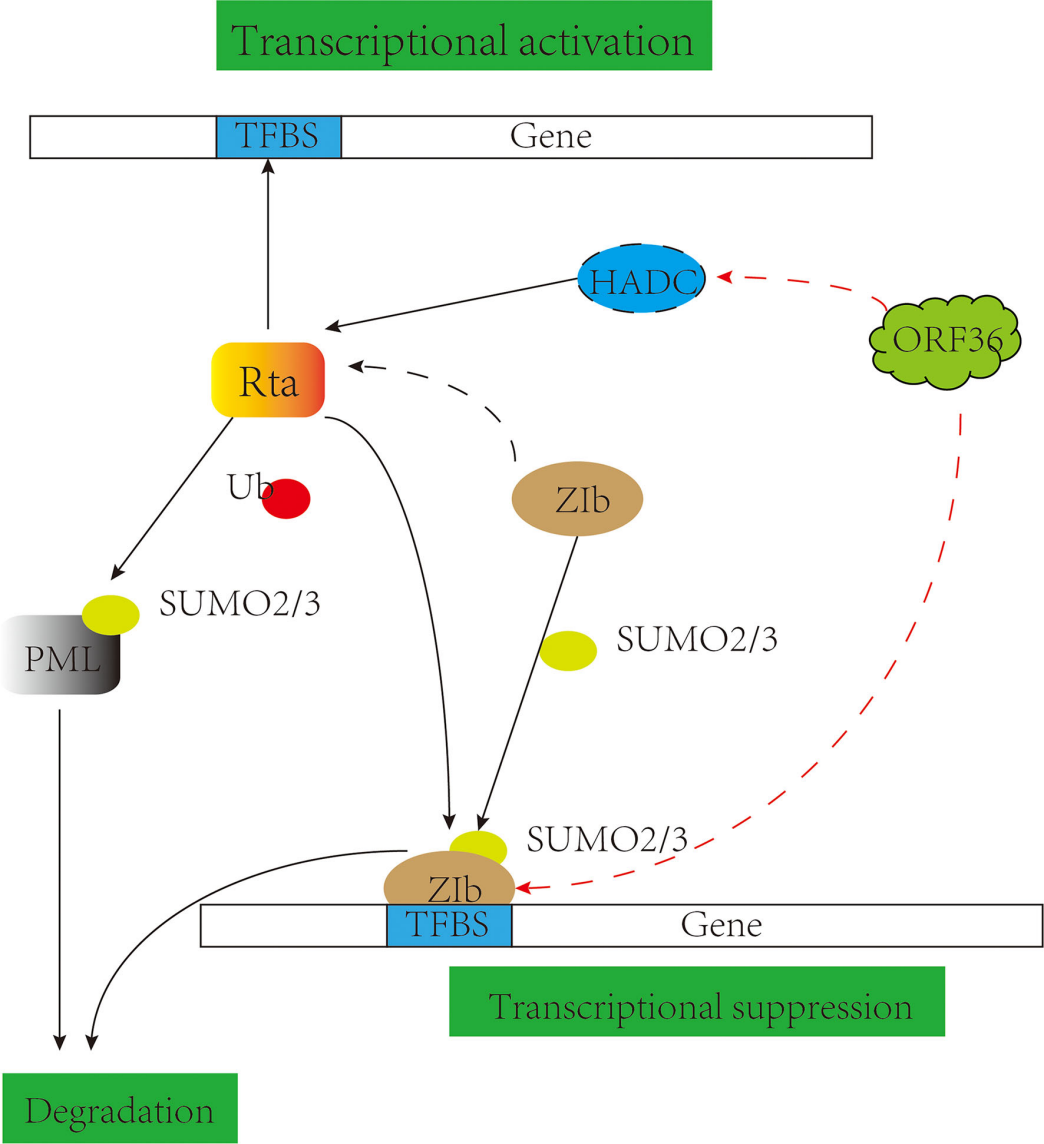
pUL13 works with SUMO proteins to promote viral replication. Gammaherpesvirus protein kinase pORF36 interacts with histone deacetylase 1 and 2 (HADC1/2) and prevents the association of these HDACs with the viral promoter driving expression of KSHV replication and transcription activator (K-Rta). pORF36 phosphorylates Thr111 of KSHV basic region-leucine zipper (K-bZIP) and inhibits the SUMO level of K-bZIP to repress the transcriptional inhibition activity.

Small Ubiquitin-related Modifier (SUMO) is a post-translational modifier protein. The SUMO system is essential in herpes virus replication, such as KSHV replication and transcription activator (K-Rta) and HSV-1 ICP0 degrade SUMO-modified promyelocytic leukemia-nuclear bodies (PML-NBs) (155, 156), inhibition of the NF-κB signaling pathway (157) and participation in degradation of IRF-3 and IRF-7 (158–160). KSHV basic region-leucine zipper (K-bZIP) is a potent transcriptional repressor that binds directly to K-Rta and attenuates K-Rta-mediated trans-activation activity, relying on SUMO modifications to regulate viral and host gene expression (161, 162). Studies have shown that KSHV ORF36 phosphorylates Thr111 of K-bZIP and inhibits the SUMO level of bZIP, causing a decrease in transcriptional inhibition activity (163) (**Figure 5**), and appears to cooperate with K-Rta inhibition of K-bZIP to promote viral transcriptional expression (164); Also involved in the phosphorylation of the cell chromatin remodeling molecule KAP-1 inhibits SUMO level and thus inhibits chromosomal remodeling capacity (165). It is also reflected in the EBV pBGLF4 negatively regulating SUMO-modified Zta to promote the establishment of viral latency (166, 167). It suggests that although the protein kinase of the γ-herpes virus cannot be modified by SUMO, its phosphorylation and SUMO can cooperate to promote viral replication.

### pUL13 promotes viral replication in conjunction with ICP22 and VP22

The interaction between herpesvirus protein kinase and viral proteins to promote its replication is a complex network, such as the interaction of KSHV pORF36 and pORF45 (168), HSV-1 pUL13 and pUL41 (169). As early as 1993, Purves reported that pUL13 phosphorylation modulated ICP22 to stabilize to increase transcription of specific subpopulations of viral RNA and accumulate corresponding viral proteins (170). Subsequently, it was found that ICP22 and pUL13 were jointly involved in phosphorylation of RNA Pol II, mediating the degradation of cyclins A and B1 and activating cdc2, in which activated cdc2 and viral DNA synthesis factor pUL42 formed a complex to recruit topoisomerase II to promote the expression of advanced genes (171–179), indicating that ICP22 and pUL13 were necessary for early gene expression of herpes virus.

In HSV-1-infected cells, UL13 protein kinase promotes the dissociation of VP22 from virions and phosphorylate VP22 (169, 180). VP22 released into cells can interact with Template-activating factor I (TAF-1) proteins and histone H4 (Histone H4), inhibit the assembly of nucleosomes on DNA and H4 histone acetylation and participate in chromatin recombination, cell cycle control, and gene regulation (181, 182). The expression of VP22 can also inhibit cGAS activity and affect natural immunity (183). It can be seen that pUL13 can promote viral replication by regulating the ICP22 and VP22 proteins and collaborating.

pUL13 is involved in multiple processes of herpes virus replication, including gene replication, transcription, and translation of viruses (184); pUL13 in herpesvirus can destroy LaminA/C to promote capsid exodus from the nucleus (185–187); assembly, maturation, and release of virions (188). It is meaningful to construct pUL13 protein interaction networks to understand better the function of UL13 protein kinases in the life cycle of the herpes virus.

### The role of pUL13 in latent infection

Induction and escape of herpesvirus genomic silencing is a biological marker of the herpes virus. Many reports suggest that pUL13 may play an essential role in the latent infection of the herpes virus. Firstly, Jolien Van Cleemput’s study found that pUL13 may be indirectly involved in the latent infection reactivation of α herpesvirus by phosphorylating other cortical proteins (189); Secondly, in γ herpes virus, EBV pBGLF4 and KSHV pORF36 are closely associated with latent infection-related proteins as such Rta, Zta, the latency-associated nuclear antigen (LANA), and TAT interacting protein 60 kD (TIP60) (190–195); Lastly, MHV-68 pORF36 inhibits the antiviral effects of bone marrow-specific STAT1 expression and promotes the establishment of latent infection of MHV-68 in spleen B cells (196). In addition, herpesvirus CHPKs can also use CD8+ T cells and many host proteins (UXT, H2AX, small ubiquitin-related modification regulatory proteins) to promote the establishment of latent infections (197–199). Although the complex mechanism of establishment and reactivation of herpes virus latent infection is unknown, UL13 protein kinase will be an essential breakthrough for the follow-up study of latent infection of herpes virus.

## Summary and prospect

pUL13 acts as a serine/threonine kinase encoded by the herpes virus. It is retained in the continuous natural screening of the virus and plays a vital role in the physiological activity of the herpes virus.

In terms of immune escape, to evade innate immune defense line and persist in host cells, pUL13 and its homologs directly or indirectly play a role in signaling pathways, which acts on different immunoregulatory proteins and many antiviral factors. Then pUL13 use varieties transcription factors and translation factors in host cells to assist the lytic cycle, such as EF-1б, H2AX, SP1, embodied in lacking pUL13 will lead to the weakening of the replication ability and virulence of the virus. At the same time, herpesvirus can use pUL13 to assist in the establishment and reactivation of its latent infection.

pUL13 can phosphorylate many protein targets and participate in the activation and inhibition of related protein functions. It is similar to a switch in the life cycle of the herpes virus. It is committed to building a systematic protein interaction network diagram of pUL13, which is conducive to unveiling pUL13 in the life cycle of the herpes virus.

Herpesvirus pUL13 is an important target for developing anti-herpesvirus drugs. With the initial clinical application of GCV (200), followed by the anti-herpesvirus trials of compounds such as Maribavir (201), K252A (202), ISIS 1082 (203, 204), and 17-DMAG (205), as well as the continuous innovation of UL13 gene deletion vaccine (206, 207) and immunotherapy (208–210). However, given that low homology among different CHPK members complicates the development of compounds targeting an entire group, further development of more broad-spectrum, efficient and safe herpesvirus protein kinase inhibitors for the treatment of herpesvirus is needed.

pUL13 undertakes a variety of functions in the life cycle of herpes virus, and exploring the mechanism of action of pUL13 can not only solve the problem of infection, transmission, and immune escape mechanism of herpes virus but also provide a theoretical basis for the research and development of clinical drugs for the anti-herpes virus.

## Author contributions

LZ and AC contributed to the design of the manuscript. XO, DS, SM, JH, QY, YW, SC, SZ, and DZ provided ideas contributing to the conception of this manuscript. RJ, ML, X-XZ, QG, and BT helped to create the figures. MW modified the manuscript. All the authors reviewed the manuscript. All authors contributed to the article and approved the submitted version.

## Funding

This work was supported by the China Agricultural Research System of MOF and MARA and Sichuan Veterinary Medicine and Drug Innovation Group of China Agricultural Research System (SCCXTD-2020-18).

## Conflict of interest

The authors declare that the research was conducted in the absence of any commercial or financial relationships that could be construed as a potential conflict of interest.

## Publisher’s note

All claims expressed in this article are solely those of the authors and do not necessarily represent those of their affiliated organizations, or those of the publisher, the editors and the reviewers. Any product that may be evaluated in this article, or claim that may be made by its manufacturer, is not guaranteed or endorsed by the publisher.

## References

[1] Qi X Yang X Cheng A Wang M Zhu D Jia R. Quantitative analysis of virulent duck enteritis virus loads in experimentally infected ducklings. Avian Dis (2008) 52(2):338–44. doi: 10.1637/8120-100207-ResNote.1 18646467

[2] Wu Y Cheng A Wang M Yang Q Zhu D Jia R. Complete genomic sequence of Chinese virulent duck enteritis virus. J Virol (2012) 86(10):5965. doi: 10.1128/jvi.00529-12 22532531 PMC3347254

[3] Jia R Cheng A Wang M Qi X Zhu D Ge H. Development and evaluation of an antigen-capture Elisa for detection of the Ul24 antigen of the duck enteritis virus, based on a polyclonal antibody against the Ul24 expression protein. J Virol Methods (2009) 161(1):38–43. doi: 10.1016/j.jviromet.2009.05.011 19467266 PMC7112936

[4] Gong T Liu L Jiang W Zhou R. Damp-sensing receptors in sterile inflammation and inflammatory diseases. Nat Rev Immunol (2020) 20(2):95–112. doi: 10.1038/s41577-019-0215-7 31558839

[5] Sun L Wu J Du F Chen X Chen ZJ. Cyclic gmp-amp synthase is a cytosolic DNA sensor that activates the type I interferon pathway. Science (2013) 339(6121):786–91. doi: 10.1126/science.1232458 PMC3863629 23258413

[6] Wu J Sun L Chen X Du F Shi H Chen C. Cyclic gmp-amp is an endogenous second messenger in innate immune signaling by cytosolic DNA. Science (2013) 339(6121):826–30. doi: 10.1126/science.1229963 PMC3855410 23258412

[7] Hayden MS Ghosh S. Nf-Kb in immunobiology. Cell Res (2011) 21(2):223–44. doi: 10.1038/cr.2011.13 PMC3193440 21243012

[8] Lawrence T. The nuclear factor nf-kappab pathway in inflammation. Cold Spring Harb Perspect Biol (2009) 1(6):a001651. doi: 10.1101/cshperspect.a001651 20457564 PMC2882124

[9] Asai R Kato A Kato K Kanamori-Koyama M Sugimoto K Sairenji T. Epstein-Barr Virus protein kinase Bglf4 is a virion tegument protein that dissociates from virions in a phosphorylation-dependent process and phosphorylates the viral immediate-early protein Bzlf1. J Virol (2006) 80(11):5125–34. doi: 10.1128/jvi.02674-05 PMC1472150 16698993

[10] Overton HA McMillan DJ Klavinskis LS Hope L Ritchie AJ Wong-kai-in P. Herpes simplex virus type 1 gene Ul13 encodes a phosphoprotein that is a component of the virion. Virology (1992) 190(1):184–92. doi: 10.1016/0042-6822(92)91204-8 1326802

[11] van Zeijl M Fairhurst J Baum EZ Sun L Jones TR. The human cytomegalovirus Ul97 protein is phosphorylated and a component of virions. Virology (1997) 231(1):72–80. doi: 10.1006/viro.1997.8523 9143304

[12] Hanks SK Quinn AM Hunter T. The protein kinase family: Conserved features and deduced phylogeny of the catalytic domains. Science (1988) 241(4861):42–52. doi: 10.1126/science.3291115 3291115

[13] Hanks SK Quinn AM. Protein kinase catalytic domain sequence database: Identification of conserved features of primary structure and classification of family members. Methods Enzymol (1991) 200:38–62. doi: 10.1016/0076-6879(91)00126-h 1956325

[14] Hanks SK Hunter T. Protein kinases 6. the eukaryotic protein kinase superfamily: Kinase (Catalytic) domain structure and classification. FASEB J (1995) 9(8):576–96. doi: 10.1096/fasebj.9.8.7768349 7768349

[15] Leader DP. Viral protein kinases and protein phosphatases. Pharmacol Ther (1993) 59(3):343–89. doi: 10.1016/0163-7258(93)90075-o 8309996

[16] Cano-Monreal GL Tavis JE Morrison LA. Substrate specificity of the herpes simplex virus type 2 Ul13 protein kinase. Virology (2008) 374(1):1–10. doi: 10.1016/j.virol.2007.11.023 18207213 PMC2396491

[17] Baek MC Krosky PM Coen DM. Relationship between autophosphorylation and phosphorylation of exogenous substrates by the human cytomegalovirus Ul97 protein kinase. J Virol (2002) 76(23):11943–52. doi: 10.1128/jvi.76.23.11943-11952.2002 PMC136897 12414936

[18] Kenyon TK Grose C. Vzv Orf47 serine protein kinase and its viral substrates. Curr Top Microbiol Immunol (2010) 342:99–111. doi: 10.1007/82_2009_5 20186612

[19] Soong W Schultz JC Patera AC Sommer MH Cohen JI. Infection of human T lymphocytes with varicella-zoster virus: An analysis with viral mutants and clinical isolates. J Virol (2000) 74(4):1864–70. doi: 10.1128/jvi.74.4.1864-1870.2000 PMC111664 10644359

[20] Besser J Sommer MH Zerboni L Bagowski CP Ito H Moffat J. Differentiation of varicella-zoster virus Orf47 protein kinase and Ie62 protein binding domains and their contributions to replication in human skin xenografts in the scid-hu mouse. J Virol (2003) 77(10):5964–74. doi: 10.1128/jvi.77.10.5964-5974.2003 PMC154036 12719588

[21] Anders PM Montgomery ND Montgomery SA Bhatt AP Dittmer DP Damania B. Human herpesvirus-encoded kinase induces b cell lymphomas in vivo. J Clin Invest (2018) 128(6):2519–34. doi: 10.1172/jci97053 PMC5983348 29733294

[22] Chen X Shan T Sun D Zhai H Dong S Kong N. Host zinc-finger cchc-type containing protein 3 inhibits pseudorabies virus proliferation by regulating type I interferon signaling. Gene (2022) 827:146480. doi: 10.1016/j.gene.2022.146480 35390445

[23] McKenzie J Lopez-Giraldez F Delecluse HJ Walsh A El-Guindy A. The Epstein-Barr virus immunoevasins Bcrf1 and Bplf1 are expressed by a mechanism independent of the canonical late pre-initiation complex. PloS Pathog (2016) 12(11):e1006008. doi: 10.1371/journal.ppat.1006008 27855219 PMC5113994

[24] Koyanagi N Kato A Takeshima K Maruzuru Y Kozuka-Hata H Oyama M. Regulation of herpes simplex virus 2 protein kinase Ul13 by phosphorylation and its role in viral pathogenesis. J Virol (2018) 92(17):e00807-18. doi: 10.1128/jvi.00807-18 29899106 PMC6096820

[25] Hu X Wang M Chen S Jia R Zhu D Liu M. The duck enteritis virus early protein, Ul13, found in both nucleus and cytoplasm, influences viral replication in cell culture. Poult Sci (2017) 96(8):2899–907. doi: 10.3382/ps/pex043 28371814

[26] Kuny CV Chinchilla K Culbertson MR Kalejta RF. Cyclin-dependent kinase-like function is shared by the beta- and gamma- subset of the conserved herpesvirus protein kinases. PloS Pathog (2010) 6(9):e1001092. doi: 10.1371/journal.ppat.1001092 20838604 PMC2936540

[27] Iwahori S Hakki M Chou S Kalejta RF. Molecular determinants for the inactivation of the retinoblastoma tumor suppressor by the viral cyclin-dependent kinase Ul97. J Biol Chem (2015) 290(32):19666–80. doi: 10.1074/jbc.M115.660043 PMC4528131 26100623

[28] Wang JT Doong SL Teng SC Lee CP Tsai CH Chen MR. Epstein-Barr Virus Bglf4 kinase suppresses the interferon regulatory factor 3 signaling pathway. J Virol (2009) 83(4):1856–69. doi: 10.1128/jvi.01099-08 PMC2643756 19052084

[29] Vandevenne P Lebrun M El Mjiyad N Ote I Di Valentin E Habraken Y. The varicella-zoster virus Orf47 kinase interferes with host innate immune response by inhibiting the activation of Irf3. PloS One (2011) 6(2):e16870. doi: 10.1371/journal.pone.0016870 21347389 PMC3036730

[30] Hwang S Kim KS Flano E Wu TT Tong LM Park AN. Conserved herpesviral kinase promotes viral persistence by inhibiting the irf-3-Mediated type I interferon response. Cell Host Microbe (2009) 5(2):166–78. doi: 10.1016/j.chom.2008.12.013 PMC2749518 19218087

[31] Bo Z Miao Y Xi R Zhong Q Bao C Chen H. Prv Ul13 inhibits cgas-Sting-Mediated ifn-B production by phosphorylating Irf3. Vet Res (2020) 51(1):118. doi: 10.1186/s13567-020-00843-4 32933581 PMC7493860

[32] Escalante CR Nistal-Villán E Shen L García-Sastre A Aggarwal AK. Structure of irf-3 bound to the prdiii-I regulatory element of the human interferon-beta enhancer. Mol Cell (2007) 26(5):703–16. doi: 10.1016/j.molcel.2007.04.022 17560375

[33] Lv L Cao M Bai J Jin L Wang X Gao Y. Prv-encoded Ul13 protein kinase acts as an antagonist of innate immunity by targeting Irf3-signaling pathways. Vet Microbiol (2020) 250:108860. doi: 10.1016/j.vetmic.2020.108860 33045632

[34] Kong Z Yin H Wang F Liu Z Luan X Sun L. Pseudorabies virus tegument protein Ul13 recruits Rnf5 to inhibit sting-mediated antiviral immunity. PloS Pathog (2022) 18(5):e1010544. doi: 10.1371/journal.ppat.1010544 35584187 PMC9154183

[35] Lv L Bai J Gao Y Jin L Wang X Cao M. Peroxiredoxin 1 interacts with Tbk1/Ikkϵ and negatively regulates pseudorabies virus propagation by promoting innate immunity. J Virol (2021) 95(19):e0092321. doi: 10.1128/jvi.00923-21 34260286 PMC8428385

[36] Fang M Zhang A Du Y Lu W Wang J Minze LJ. Trim18 is a critical regulator of viral myocarditis and organ inflammation. J BioMed Sci (2022) 29(1):55. doi: 10.1186/s12929-022-00840-z 35909127 PMC9339186

[37] Li Q Lin L Tong Y Liu Y Mou J Wang X. Trim29 negatively controls antiviral immune response through targeting sting for degradation. Cell Discovery (2018) 4:13. doi: 10.1038/s41421-018-0010-9 29581886 PMC5859251

[38] Alexopoulou L Holt AC Medzhitov R Flavell RA. Recognition of double-stranded rna and activation of nf-kappab by toll-like receptor 3. Nature (2001) 413(6857):732–8. doi: 10.1038/35099560 11607032

[39] Kawai T Akira S. Signaling to nf-kappab by toll-like receptors. Trends Mol Med (2007) 13(11):460–9. doi: 10.1016/j.molmed.2007.09.002 18029230

[40] Chang LS Wang JT Doong SL Lee CP Chang CW Tsai CH. Epstein-Barr Virus Bglf4 kinase downregulates nf-Kb transactivation through phosphorylation of coactivator uxt. J Virol (2012) 86(22):12176–86. doi: 10.1128/jvi.01918-12 PMC3486492 22933289

[41] Mounce BC Mboko WP Bigley TM Terhune SS Tarakanova VL. A conserved gammaherpesvirus protein kinase targets histone deacetylases 1 and 2 to facilitate viral replication in primary macrophages. J Virol (2013) 87(13):7314–25. doi: 10.1128/jvi.02713-12 PMC3700300 23616648

[42] Dong X Feng P. Murine gamma herpesvirus 68 hijacks mavs and ikkβ to abrogate nfκb activation and antiviral cytokine production. PloS Pathog (2011) 7(11):e1002336. doi: 10.1371/journal.ppat.1002336 22110409 PMC3213086

[43] Schaller AM Tucker J Willis I Glaunsinger BA. Conserved herpesvirus kinase Orf36 activates B2 retrotransposons during murine gammaherpesvirus infection. J Virol (2020) 94(14):e00262-20. doi: 10.1128/jvi.00262-20 32404524 PMC7343202

[44] Kassube SA Fang J Grob P Yakovchuk P Goodrich JA Nogales E. Structural insights into transcriptional repression by noncoding rnas that bind to human pol ii. J Mol Biol (2013) 425(19):3639–48. doi: 10.1016/j.jmb.2012.08.024 PMC3556225 22954660

[45] Ponicsan SL Houel S Old WM Ahn NG Goodrich JA Kugel JF. The non-coding B2 rna binds to the DNA cleft and active-site region of rna polymerase ii. J Mol Biol (2013) 425(19):3625–38. doi: 10.1016/j.jmb.2013.01.035 PMC3672349 23416138

[46] Darnell JE Jr. Kerr IM Stark GR. Jak-stat pathways and transcriptional activation in response to ifns and other extracellular signaling proteins. Science (1994) 264(5164):1415–21. doi: 10.1126/science.8197455 8197455

[47] Stark GR Darnell JE Jr. The jak-stat pathway at twenty. Immunity (2012) 36(4):503–14. doi: 10.1016/j.immuni.2012.03.013 PMC3909993 22520844

[48] Sato Y Koshizuka T Ishibashi K Hashimoto K Ishioka K Ikuta K. Involvement of herpes simplex virus type 1 Ul13 protein kinase in induction of socs genes, the negative regulators of cytokine signaling. Microbiol Immunol (2017) 61(5):159–67. doi: 10.1111/1348-0421.12483 28419615

[49] Vizcaíno C Mansilla S Portugal J. Sp1 transcription factor: A long-standing target in cancer chemotherapy. Pharmacol Ther (2015) 152:111–24. doi: 10.1016/j.pharmthera.2015.05.008 25960131

[50] Barclay JL Anderson ST Waters MJ Curlewis JD. Socs3 as a tumor suppressor in breast cancer cells, and its regulation by prl. Int J Cancer (2009) 124(8):1756–66. doi: 10.1002/ijc.24172 19115200

[51] Yokota S Yokosawa N Okabayashi T Suzutani T Miura S Jimbow K. Induction of suppressor of cytokine signaling-3 by herpes simplex virus type 1 contributes to inhibition of the interferon signaling pathway. J Virol (2004) 78(12):6282–6. doi: 10.1128/jvi.78.12.6282-6286.2004 PMC416529 15163721

[52] Yokota S Yokosawa N Okabayashi T Suzutani T Fujii N. Induction of suppressor of cytokine signaling-3 by herpes simplex virus type 1 confers efficient viral replication. Virology (2005) 338(1):173–81. doi: 10.1016/j.virol.2005.04.028 15939448

[53] Shen X Hong F Nguyen VA Gao B. Il-10 attenuates ifn-Alpha-Activated Stat1 in the liver: Involvement of Socs2 and Socs3. FEBS Lett (2000) 480(2-3):132–6. doi: 10.1016/s0014-5793(00)01905-0 11034314

[54] Jones KA Tjian R. Sp1 binds to promoter sequences and activates herpes simplex virus 'Immediate-early' gene transcription in vitro. Nature (1985) 317(6033):179–82. doi: 10.1038/317179a0 2993923

[55] Ostler JB Thunuguntla P Hendrickson BY Jones C. Transactivation of herpes simplex virus 1 (Hsv-1) infected cell protein 4 enhancer by glucocorticoid receptor and stress-induced transcription factors requires overlapping krüppel-like transcription factor 4/Sp1 binding sites. J Virol (2021) 95(4):e01776-20. doi: 10.1128/jvi.01776-20 33208447 PMC7851558

[56] Gu B Rivera-Gonzalez R Smith CA DeLuca NA. Herpes simplex virus infected cell polypeptide 4 preferentially represses Sp1-activated over basal transcription from its own promoter. Proc Natl Acad Sci U.S.A. (1993) 90(20):9528–32. doi: 10.1073/pnas.90.20.9528 PMC47602 8415735

[57] Kim DB DeLuca NA. Phosphorylation of transcription factor Sp1 during herpes simplex virus type 1 infection. J Virol (2002) 76(13):6473–9. doi: 10.1128/jvi.76.13.6473-6479.2002 PMC136260 12050359

[58] Akhtar LN Benveniste EN. Viral exploitation of host socs protein functions. J Virol (2011) 85(5):1912–21. doi: 10.1128/jvi.01857-10 PMC3067810 21084484

[59] Choi EJ Lee CH Shin OS. Suppressor of cytokine signaling 3 expression induced by varicella-zoster virus infection results in the modulation of virus replication. Scand J Immunol (2015) 82(4):337–44. doi: 10.1111/sji.12323 26072679

[60] Frey KG Ahmed CM Dabelic R Jager LD Noon-Song EN Haider SM. Hsv-1-Induced socs-1 expression in keratinocytes: Use of a socs-1 antagonist to block a novel mechanism of viral immune evasion. J Immunol (2009) 183(2):1253–62. doi: 10.4049/jimmunol.0900570 PMC2706942 19542368

[61] Michaud F Coulombe F Gaudreault E Paquet-Bouchard C Rola-Pleszczynski M Gosselin J. Epstein-Barr Virus interferes with the amplification of ifnalpha secretion by activating suppressor of cytokine signaling 3 in primary human monocytes. PloS One (2010) 5(7):e11908. doi: 10.1371/journal.pone.0011908 20689596 PMC2912847

[62] Sasaki A Yasukawa H Suzuki A Kamizono S Syoda T Kinjyo I. Cytokine-inducible Sh2 protein-3 (Cis3/Socs3) inhibits janus tyrosine kinase by binding through the n-terminal kinase inhibitory region as well as Sh2 domain. Genes Cells (1999) 4(6):339–51. doi: 10.1046/j.1365-2443.1999.00263.x 10421843

[63] Yasukawa H Misawa H Sakamoto H Masuhara M Sasaki A Wakioka T. The jak-binding protein jab inhibits janus tyrosine kinase activity through binding in the activation loop. EMBO J (1999) 18(5):1309–20. doi: 10.1093/emboj/18.5.1309 PMC1171221 10064597

[64] Wormald S Zhang JG Krebs DL Mielke LA Silver J Alexander WS. The comparative roles of suppressor of cytokine signaling-1 and -3 in the inhibition and desensitization of cytokine signaling. J Biol Chem (2006) 281(16):11135–43. doi: 10.1074/jbc.M509595200 16473883

[65] Wang Y van Boxel-Dezaire AH Cheon H Yang J Stark GR. Stat3 activation in response to il-6 is prolonged by the binding of il-6 receptor to egf receptor. Proc Natl Acad Sci U.S.A. (2013) 110(42):16975–80. doi: 10.1073/pnas.1315862110 PMC3801081 24082147

[66] Carow B Rottenberg ME. Socs3, a major regulator of infection and inflammation. Front Immunol (2014) 5:58. doi: 10.3389/fimmu.2014.00058 24600449 PMC3928676

[67] Karlsen AE Heding PE Frobøse H Rønn SG Kruhøffer M Orntoft TF. Suppressor of cytokine signalling (Socs)-3 protects beta cells against il-1beta-Mediated toxicity through inhibition of multiple nuclear factor-Kappab-Regulated proapoptotic pathways. Diabetologia (2004) 47(11):1998–2011. doi: 10.1007/s00125-004-1568-3 15578154

[68] Zamanian-Daryoush M Mogensen TH DiDonato JA Williams BR. Nf-kappab activation by double-Stranded-Rna-Activated protein kinase (Pkr) is mediated through nf-Kappab-Inducing kinase and ikappab kinase. Mol Cell Biol (2000) 20(4):1278–90. doi: 10.1128/mcb.20.4.1278-1290.2000 PMC85265 10648614

[69] Gu L Ge Z Wang Y Shen M Zhao P Chen W. Double-stranded rna-dependent kinase pkr activates nf-Kb pathway in acute pancreatitis. Biochem Biophys Res Commun (2018) 503(3):1563–9. doi: 10.1016/j.bbrc.2018.07.080 30031606

[70] Paludan SR Mogensen SC. Virus-cell interactions regulating induction of tumor necrosis factor alpha production in macrophages infected with herpes simplex virus. J Virol (2001) 75(21):10170–8. doi: 10.1128/jvi.75.21.10170-10178.2001 PMC114591 11581385

[71] Hu Z Du H Lin G Han K Cheng X Feng Z. Grass carp (Ctenopharyngodon idella) pact induces cell apoptosis and activates nf-Кb *Via* pkr. Fish Shellfish Immunol (2020) 103:377–84. doi: 10.1016/j.fsi.2020.05.036 32454210

[72] Li X Wu Z An X Mei Q Bai M Hanski L. Blockade of the Lrp16-Pkr-Nf-Kb signaling axis sensitizes colorectal carcinoma cells to DNA-damaging cytotoxic therapy. Elife (2017) 6 :e27301. doi: 10.7554/eLife.27301 28820388 PMC5562444

[73] Gil J Alcamí J Esteban M. Activation of nf-kappa b by the dsrna-dependent protein kinase, pkr involves the I kappa b kinase complex. Oncogene (2000) 19(11):1369–78. doi: 10.1038/sj.onc.1203448 10723127

[74] Schulz O Pichlmair A Rehwinkel J Rogers NC Scheuner D Kato H. Protein kinase r contributes to immunity against specific viruses by regulating interferon mrna integrity. Cell Host Microbe (2010) 7(5):354–61. doi: 10.1016/j.chom.2010.04.007 PMC2919169 20478537

[75] Yoon CH Lee ES Lim DS Bae YS. Pkr, a P53 target gene, plays a crucial role in the tumor-suppressor function of P53. Proc Natl Acad Sci U.S.A. (2009) 106(19):7852–7. doi: 10.1073/pnas.0812148106 PMC2683089 19416861

[76] Cuddihy AR Wong AH Tam NW Li S Koromilas AE. The double-stranded rna activated protein kinase pkr physically associates with the tumor suppressor P53 protein and phosphorylates human P53 on serine 392 in vitro. Oncogene (1999) 18(17):2690–702. doi: 10.1038/sj.onc.1202620 10348343

[77] Huang Q Xie D Mao H Wang H Wu Z Huang K. Ctenopharyngodon idella P53 mediates between nf-Kb and pkr at the transcriptional level. Fish Shellfish Immunol (2017) 69:258–64. doi: 10.1016/j.fsi.2017.08.012 28818618

[78] Dey M Cao C Dar AC Tamura T Ozato K Sicheri F. Mechanistic link between pkr dimerization, autophosphorylation, and Eif2alpha substrate recognition. Cell (2005) 122(6):901–13. doi: 10.1016/j.cell.2005.06.041 16179259

[79] Dar AC Dever TE Sicheri F. Higher-order substrate recognition of Eif2alpha by the rna-dependent protein kinase pkr. Cell (2005) 122(6):887–900. doi: 10.1016/j.cell.2005.06.044 16179258

[80] Su Q Wang S Baltzis D Qu LK Wong AH Koromilas AE. Tyrosine phosphorylation acts as a molecular switch to full-scale activation of the Eif2alpha rna-dependent protein kinase. Proc Natl Acad Sci U.S.A. (2006) 103(1):63–8. doi: 10.1073/pnas.0508207103 PMC1324992 16373505

[81] Rabouw HH Langereis MA Knaap RC Dalebout TJ Canton J Sola I. Middle East respiratory coronavirus accessory protein 4a inhibits pkr-mediated antiviral stress responses. PloS Pathog (2016) 12(10):e1005982. doi: 10.1371/journal.ppat.1005982 27783669 PMC5081173

[82] Sharma NR Majerciak V Kruhlak MJ Zheng ZM. Kshv inhibits stress granule formation by viral Orf57 blocking pkr activation. PloS Pathog (2017) 13(10):e1006677. doi: 10.1371/journal.ppat.1006677 29084250 PMC5679657

[83] Dauber B Pelletier J Smiley JR. The herpes simplex virus 1 vhs protein enhances translation of viral true late mrnas and virus production in a cell type-dependent manner. J Virol (2011) 85(11):5363–73. doi: 10.1128/jvi.00115-11 PMC3094992 21430045

[84] Dauber B Poon D Dos Santos T Duguay BA Mehta N Saffran HA. The herpes simplex virus virion host shutoff protein enhances translation of viral true late mrnas independently of suppressing protein kinase r and stress granule formation. J Virol (2016) 90(13):6049–57. doi: 10.1128/jvi.03180-15 PMC4907241 27099317

[85] Li C Zhu Z Du X Cao W Yang F Zhang X. Foot-and-Mouth disease virus induces lysosomal degradation of host protein kinase pkr by 3c proteinase to facilitate virus replication. Virology (2017) 509:222–31. doi: 10.1016/j.virol.2017.06.023 PMC7126777 28662438

[86] Valchanova RS Picard-Maureau M Budt M Brune W. Murine cytomegalovirus M142 and M143 are both required to block protein kinase r-mediated shutdown of protein synthesis. J Virol (2006) 80(20):10181–90. doi: 10.1128/jvi.00908-06 PMC1617306 17005695

[87] McKenna SA Lindhout DA Shimoike T Aitken CE Puglisi JD. Viral dsrna inhibitors prevent self-association and autophosphorylation of pkr. J Mol Biol (2007) 372(1):103–13. doi: 10.1016/j.jmb.2007.06.028 PMC3710116 17619024

[88] Poppers J Mulvey M Perez C Khoo D Mohr I. Identification of a lytic-cycle Epstein-Barr virus gene product that can regulate pkr activation. J Virol (2003) 77(1):228–36. doi: 10.1128/jvi.77.1.228-236.2003 PMC140577 12477828

[89] Lussignol M Queval C Bernet-Camard MF Cotte-Laffitte J Beau I Codogno P. The herpes simplex virus 1 Us11 protein inhibits autophagy through its interaction with the protein kinase pkr. J Virol (2013) 87(2):859–71. doi: 10.1128/jvi.01158-12 PMC3554085 23115300

[90] He B Gross M Roizman B. The Gamma134.5 protein of herpes simplex virus 1 has the structural and functional attributes of a protein phosphatase 1 regulatory subunit and is present in a high molecular weight complex with the enzyme in infected cells. J Biol Chem (1998) 273(33):20737–43. doi: 10.1074/jbc.273.33.20737 9694816

[91] Esteban M García MA Domingo-Gil E Arroyo J Nombela C Rivas C. The latency protein Lana2 from kaposi's sarcoma-associated herpesvirus inhibits apoptosis induced by dsrna-activated protein kinase but not rnase l activation. J Gen Virol (2003) 84(Pt 6):1463–70. doi: 10.1099/vir.0.19014-0 12771415

[92] He B Gross M Roizman B. The Gamma(1)34.5 protein of herpes simplex virus 1 complexes with protein phosphatase 1alpha to dephosphorylate the alpha subunit of the eukaryotic translation initiation factor 2 and preclude the shutoff of protein synthesis by double-stranded rna-activated protein kinase. Proc Natl Acad Sci U.S.A. (1997) 94(3):843–8. doi: 10.1073/pnas.94.3.843 PMC19601 9023344

[93] Borghese F Michiels T. The leader protein of cardioviruses inhibits stress granule assembly. J Virol (2011) 85(18):9614–22. doi: 10.1128/jvi.00480-11 PMC3165746 21752908

[94] Borghese F Sorgeloos F Cesaro T Michiels T. The leader protein of theiler's virus prevents the activation of pkr. J Virol (2019) 93(19):e01010-19. doi: 10.1128/jvi.01010-19 31292248 PMC6744238

[95] Pennisi R Musarra-Pizzo M Lei Z Zhou GG Sciortino MT. Vhs, Us3 and Ul13 viral tegument proteins are required for herpes simplex virus-induced modification of protein kinase r. Sci Rep (2020) 10(1):5580. doi: 10.1038/s41598-020-62619-2 32221365 PMC7101438

[96] Costa-Mattioli M Walter P. The integrated stress response: From mechanism to disease. Science (2020) 368(6489):eaat5314. doi: 10.1126/science.aat5314 32327570 PMC8997189

[97] Watanabe T Imamura T Hiasa Y. Roles of protein kinase r in cancer: Potential as a therapeutic target. Cancer Sci (2018) 109(4):919–25. doi: 10.1111/cas.13551 PMC5891186 29478262

[98] Raafat N Sadowski-Cron C Mengus C Heberer M Spagnoli GC Zajac P. Preventing vaccinia virus class-I epitopes presentation by hsv-Icp47 enhances the immunogenicity of a tap-independent cancer vaccine epitope. Int J Cancer (2012) 131(5):E659–69. doi: 10.1002/ijc.27362 22116674

[99] Aisenbrey C Sizun C Koch J Herget M Abele R Bechinger B. Structure and dynamics of membrane-associated Icp47, a viral inhibitor of the mhc I antigen-processing machinery. J Biol Chem (2006) 281(41):30365–72. doi: 10.1074/jbc.M603000200 16835230

[100] Deruelle MJ Van den Broeke C Nauwynck HJ Mettenleiter TC Favoreel HW. Pseudorabies virus Us3- and Ul49.5-dependent and -independent downregulation of mhc I cell surface expression in different cell types. Virology (2009) 395(2):172–81. doi: 10.1016/j.virol.2009.09.019 19819514

[101] Imai T Koyanagi N Ogawa R Shindo K Suenaga T Sato A. Us3 kinase encoded by herpes simplex virus 1 mediates downregulation of cell surface major histocompatibility complex class I and evasion of Cd8+ T cells. PloS One (2013) 8(8):e72050. doi: 10.1371/journal.pone.0072050 23951282 PMC3741198

[102] Koyanagi N Imai T Shindo K Sato A Fujii W Ichinohe T. Herpes simplex virus-1 evasion of Cd8+ T cell accumulation contributes to viral encephalitis. J Clin Invest (2017) 127(10):3784–95. doi: 10.1172/jci92931 PMC5617679 28891812

[103] Spranger S Dai D Horton B Gajewski TF. Tumor-residing Batf3 dendritic cells are required for effector T cell trafficking and adoptive T cell therapy. Cancer Cell (2017) 31(5):711–23.e4. doi: 10.1016/j.ccell.2017.04.003 28486109 PMC5650691

[104] Canny SP Goel G Reese TA Zhang X Xavier R Virgin HW. Latent gammaherpesvirus 68 infection induces distinct transcriptional changes in different organs. J Virol (2014) 88(1):730–8. doi: 10.1128/jvi.02708-13 PMC3911696 24155394

[105] Prabhakaran K Sheridan BS Kinchington PR Khanna KM Decman V Lathrop K. Sensory neurons regulate the effector functions of Cd8+ T cells in controlling hsv-1 latency ex vivo. Immunity (2005) 23(5):515–25. doi: 10.1016/j.immuni.2005.09.017 16286019

[106] Knickelbein JE Khanna KM Yee MB Baty CJ Kinchington PR Hendricks RL. Noncytotoxic lytic granule-mediated Cd8+ T cell inhibition of hsv-1 reactivation from neuronal latency. Science (2008) 322(5899):268–71. doi: 10.1126/science.1164164 PMC2680315 18845757

[107] Nair A Hunzeker J Bonneau RH. Modulation of microglia and Cd8(+) T cell activation during the development of stress-induced herpes simplex virus type-1 encephalitis. Brain Behav Immun (2007) 21(6):791–806. doi: 10.1016/j.bbi.2007.01.005 17349776

[108] Menendez CM Carr DJJ. Herpes simplex virus-1 infects the olfactory bulb shortly following ocular infection and exhibits a long-term inflammatory profile in the form of effector and hsv-1-Specific T cells. J Neuroinflamm (2017) 14(1):124. doi: 10.1186/s12974-017-0903-9 PMC5481928 28645309

[109] Lang A Nikolich-Zugich J. Development and migration of protective Cd8+ T cells into the nervous system following ocular herpes simplex virus-1 infection. J Immunol (2005) 174(5):2919–25. doi: 10.4049/jimmunol.174.5.2919 15728503

[110] Banerjee K Biswas PS Kumaraguru U Schoenberger SP Rouse BT. Protective and pathological roles of virus-specific and bystander Cd8+ T cells in herpetic stromal keratitis. J Immunol (2004) 173(12):7575–83. doi: 10.4049/jimmunol.173.12.7575 15585885

[111] Coquel F Silva MJ Técher H Zadorozhny K Sharma S Nieminuszczy J. Samhd1 acts at stalled replication forks to prevent interferon induction. Nature (2018) 557(7703):57–61. doi: 10.1038/s41586-018-0050-1 29670289

[112] Cabello-Lobato MJ Wang S Schmidt CK. Samhd1 sheds moonlight on DNA double-strand break repair. Trends Genet (2017) 33(12):895–7. doi: 10.1016/j.tig.2017.09.007 28969870

[113] Hollenbaugh JA Gee P Baker J Daly MB Amie SM Tate J. Host factor Samhd1 restricts DNA viruses in non-dividing myeloid cells. PloS Pathog (2013) 9(6):e1003481. doi: 10.1371/journal.ppat.1003481 23825958 PMC3694861

[114] Kim ET White TE Brandariz-Núñez A Diaz-Griffero F Weitzman MD. Samhd1 restricts herpes simplex virus 1 in macrophages by limiting DNA replication. J Virol (2013) 87(23):12949–56. doi: 10.1128/jvi.02291-13 PMC3838123 24067963

[115] Sze A Belgnaoui SM Olagnier D Lin R Hiscott J van Grevenynghe J. Host restriction factor Samhd1 limits human T cell leukemia virus type 1 infection of monocytes *Via* sting-mediated apoptosis. Cell Host Microbe (2013) 14(4):422–34. doi: 10.1016/j.chom.2013.09.009 24139400

[116] Chen Z Zhu M Pan X Zhu Y Yan H Jiang T. Inhibition of hepatitis b virus replication by Samhd1. Biochem Biophys Res Commun (2014) 450(4):1462–8. doi: 10.1016/j.bbrc.2014.07.023 25019997

[117] Laguette N Sobhian B Casartelli N Ringeard M Chable-Bessia C Ségéral E. Samhd1 is the dendritic- and myeloid-Cell-Specific hiv-1 restriction factor counteracted by vpx. Nature (2011) 474(7353):654–7. doi: 10.1038/nature10117 PMC3595993 21613998

[118] Chougui G Munir-Matloob S Matkovic R Martin MM Morel M Lahouassa H. Hiv-2/Siv viral protein X counteracts hush repressor complex. Nat Microbiol (2018) 3(8):891–7. doi: 10.1038/s41564-018-0179-6 29891865

[119] Hrecka K Hao C Gierszewska M Swanson SK Kesik-Brodacka M Srivastava S. Vpx relieves inhibition of hiv-1 infection of macrophages mediated by the Samhd1 protein. Nature (2011) 474(7353):658–61. doi: 10.1038/nature10195 PMC3179858 21720370

[120] Goujon C Rivière L Jarrosson-Wuilleme L Bernaud J Rigal D Darlix JL. Sivsm/Hiv-2 vpx proteins promote retroviral escape from a proteasome-dependent restriction pathway present in human dendritic cells. Retrovirology (2007) 4:2. doi: 10.1186/1742-4690-4-2 17212817 PMC1779362

[121] Rudd SG Tsesmetzis N Sanjiv K Paulin CB Sandhow L Kutzner J. Ribonucleotide reductase inhibitors suppress Samhd1 ara-ctpase activity enhancing cytarabine efficacy. EMBO Mol Med (2020) 12(3):e10419. doi: 10.15252/emmm.201910419 31950591 PMC7059017

[122] Iwasawa C Tamura R Sugiura Y Suzuki S Kuzumaki N Narita M. Increased cytotoxicity of herpes simplex virus thymidine kinase expression in human induced pluripotent stem cells. Int J Mol Sci (2019) 20(4):810. doi: 10.3390/ijms20040810 30769780 PMC6413063

[123] Valle-Casuso JC Allouch A David A Lenzi GM Studdard L Barré-Sinoussi F. P21 restricts hiv-1 in monocyte-derived dendritic cells through the reduction of deoxynucleoside triphosphate biosynthesis and regulation of Samhd1 antiviral activity. J Virol (2017) 91(23):e01324-17. doi: 10.1128/jvi.01324-17 28931685 PMC5686764

[124] Yan J Hao C DeLucia M Swanson S Florens L Washburn MP. Cyclina2-Cyclin-Dependent kinase regulates Samhd1 protein phosphohydrolase domain. J Biol Chem (2015) 290(21):13279–92. doi: 10.1074/jbc.M115.646588 PMC4505580 25847232

[125] White TE Brandariz-Nuñez A Valle-Casuso JC Amie S Nguyen LA Kim B. The retroviral restriction ability of Samhd1, but not its deoxynucleotide triphosphohydrolase activity, is regulated by phosphorylation. Cell Host Microbe (2013) 13(4):441–51. doi: 10.1016/j.chom.2013.03.005 PMC3864637 23601106

[126] Szaniawski MA Spivak AM Cox JE Catrow JL Hanley T Williams E. Samhd1 phosphorylation coordinates the anti-Hiv-1 response by diverse interferons and tyrosine kinase inhibition. mBio (2018) 9(3):e00819-18. doi: 10.1128/mBio.00819-18 29764952 PMC5954222

[127] Businger R Deutschmann J Gruska I Milbradt J Wiebusch L Gramberg T. Human cytomegalovirus overcomes Samhd1 restriction in macrophages *Via* Pul97. Nat Microbiol (2019) 4(12):2260–72. doi: 10.1038/s41564-019-0557-8 31548682

[128] Zhang K Lv DW Li R. Conserved herpesvirus protein kinases target Samhd1 to facilitate virus replication. Cell Rep (2019) 28(2):449–59.e5. doi: 10.1016/j.celrep.2019.04.020 31291580 PMC6668718

[129] Chen S Bonifati S Qin Z St Gelais C Kodigepalli KM Barrett BS. Samhd1 suppresses innate immune responses to viral infections and inflammatory stimuli by inhibiting the nf-Kb and interferon pathways. Proc Natl Acad Sci U.S.A. (2018) 115(16):E3798–e807. doi: 10.1073/pnas.1801213115 PMC5910870 29610295

[130] Reuvers TGA Kanaar R Nonnekens J. DNA Damage-inducing anticancer therapies: From global to precision damage. Cancers (Basel) (2020) 12(8):2098. doi: 10.3390/cancers12082098 32731592 PMC7463878

[131] Jackson SP Bartek J. The DNA-damage response in human biology and disease. Nature (2009) 461(7267):1071–8. doi: 10.1038/nature08467 PMC2906700 19847258

[132] Luftig MA. Viruses and the DNA damage response: Activation and antagonism. Annu Rev Virol (2014) 1(1):605–25. doi: 10.1146/annurev-virology-031413-085548 26958736

[133] Lilley CE Carson CT Muotri AR Gage FH Weitzman MD. DNA Repair proteins affect the lifecycle of herpes simplex virus 1. Proc Natl Acad Sci U.S.A. (2005) 102(16):5844–9. doi: 10.1073/pnas.0501916102 PMC556126 15824307

[134] Wilkinson DE Weller SK. Herpes simplex virus type I disrupts the atr-dependent DNA-damage response during lytic infection. J Cell Sci (2006) 119(Pt 13):2695–703. doi: 10.1242/jcs.02981 PMC4427570 16757521

[135] Li R Zhu J Xie Z Liao G Liu J Chen MR. Conserved herpesvirus kinases target the DNA damage response pathway and Tip60 histone acetyltransferase to promote virus replication. Cell Host Microbe (2011) 10(4):390–400. doi: 10.1016/j.chom.2011.08.013 22018239 PMC3253558

[136] Yedjou CG Tchounwou HM Tchounwou PB. DNA Damage, cell cycle arrest, and apoptosis induction caused by lead in human leukemia cells. Int J Environ Res Public Health (2015) 13(1):ijerph13010056. doi: 10.3390/ijerph13010056 26703663 PMC4730447

[137] van Attikum H Gasser SM. Crosstalk between histone modifications during the DNA damage response. Trends Cell Biol (2009) 19(5):207–17. doi: 10.1016/j.tcb.2009.03.001 19342239

[138] Bogliolo M Lyakhovich A Callén E Castellà M Cappelli E Ramírez MJ. Histone H2ax and fanconi anemia Fancd2 function in the same pathway to maintain chromosome stability. EMBO J (2007) 26(5):1340–51. doi: 10.1038/sj.emboj.7601574 PMC1817623 17304220

[139] Saldivar JC Cortez D Cimprich KA. The essential kinase atr: Ensuring faithful duplication of a challenging genome. Nat Rev Mol Cell Biol (2017) 18(10):622–36. doi: 10.1038/nrm.2017.67 PMC5796526 28811666

[140] Banerjee S Smallwood A Hultén M. Atp-dependent reorganization of human sperm nuclear chromatin. J Cell Sci (1995) 108(Pt 2):755–65. doi: 10.1242/jcs.108.2.755 7769017

[141] Tarakanova VL Leung-Pineda V Hwang S Yang CW Matatall K Basson M. Gamma-herpesvirus kinase actively initiates a DNA damage response by inducing phosphorylation of H2ax to foster viral replication. Cell Host Microbe (2007) 1(4):275–86. doi: 10.1016/j.chom.2007.05.008 PMC2034359 18005708

[142] Chen MR Chang SJ Huang H Chen JY. A protein kinase activity associated with Epstein-Barr virus Bglf4 phosphorylates the viral early antigen ea-d in vitro. J Virol (2000) 74(7):3093–104. doi: 10.1128/jvi.74.7.3093-3104.2000 PMC111808 10708424

[143] Mounce BC Tsan FC Droit L Kohler S Reitsma JM Cirillo LA. Gammaherpesvirus gene expression and DNA synthesis are facilitated by viral protein kinase and histone variant H2ax. Virology (2011) 420(2):73–81. doi: 10.1016/j.virol.2011.08.019 21943826 PMC3204369

[144] Jha HC Upadhyay SK M AJP Lu J Cai Q Saha A. H2ax phosphorylation is important for Lana-mediated kaposi's sarcoma-associated herpesvirus episome persistence. J Virol (2013) 87(9):5255–69. doi: 10.1128/jvi.03575-12 PMC3624323 23449797

[145] Li R Wang L Liao G Guzzo CM Matunis MJ Zhu H. Sumo binding by the Epstein-Barr virus protein kinase Bglf4 is crucial for Bglf4 function. J Virol (2012) 86(10):5412–21. doi: 10.1128/jvi.00314-12 PMC3347263 22398289

[146] Ming X Bo Z Miao Y Chen H Bao C Sun L. Pseudorabies virus kinase Ul13 phosphorylates H2ax to foster viral replication. FASEB J (2022) 36(3):e22221. doi: 10.1096/fj.202101360RR 35199383

[147] Yamamoto T Ali MA Liu X Cohen JI. Activation of H2ax and atm in varicella-zoster virus (Vzv)-infected cells is associated with expression of specific vzv genes. Virology (2014) 452-453:52–8. doi: 10.1016/j.virol.2013.12.039 PMC4789179 24606682

[148] Daikoku T Kudoh A Sugaya Y Iwahori S Shirata N Isomura H. Postreplicative mismatch repair factors are recruited to Epstein-Barr virus replication compartments. J Biol Chem (2006) 281(16):11422–30. doi: 10.1074/jbc.M510314200 16510450

[149] Wilkinson DE Weller SK. Recruitment of cellular recombination and repair proteins to sites of herpes simplex virus type 1 DNA replication is dependent on the composition of viral proteins within prereplicative sites and correlates with the induction of the DNA damage response. J Virol (2004) 78(9):4783–96. doi: 10.1128/jvi.78.9.4783-4796.2004 PMC387708 15078960

[150] Taylor TJ Knipe DM. Proteomics of herpes simplex virus replication compartments: Association of cellular DNA replication, repair, recombination, and chromatin remodeling proteins with Icp8. J Virol (2004) 78(11):5856–66. doi: 10.1128/jvi.78.11.5856-5866.2004 PMC415816 15140983

[151] Bhatt AP Wong JP Weinberg MS Host KM Giffin LC Buijnink J. A viral kinase mimics S6 kinase to enhance cell proliferation. Proc Natl Acad Sci U.S.A. (2016) 113(28):7876–81. doi: 10.1073/pnas.1600587113 PMC4948314 27342859

[152] Kawaguchi Y Matsumura T Roizman B Hirai K. Cellular elongation factor 1delta is modified in cells infected with representative alpha-, beta-, or gammaherpesviruses. J Virol (1999) 73(5):4456–60. doi: 10.1128/jvi.73.5.4456-4460.1999 PMC104232 10196346

[153] Kato K Kawaguchi Y Tanaka M Igarashi M Yokoyama A Matsuda G. Epstein-Barr Virus-encoded protein kinase Bglf4 mediates hyperphosphorylation of cellular elongation factor 1delta (Ef-1delta): Ef-1delta is universally modified by conserved protein kinases of herpesviruses in mammalian cells. J Gen Virol (2001) 82(Pt 6):1457–63. doi: 10.1099/0022-1317-82-6-1457 11369891

[154] Kawaguchi Y Van Sant C Roizman B. Eukaryotic elongation factor 1delta is hyperphosphorylated by the protein kinase encoded by the U(L)13 gene of herpes simplex virus 1. J Virol (1998) 72(3):1731–6. doi: 10.1128/jvi.72.3.1731-1736.1998 PMC109460 9499021

[155] Boutell C Cuchet-Lourenço D Vanni E Orr A Glass M McFarlane S. A viral ubiquitin ligase has substrate preferential sumo targeted ubiquitin ligase activity that counteracts intrinsic antiviral defence. PloS Pathog (2011) 7(9):e1002245. doi: 10.1371/journal.ppat.1002245 21949651 PMC3174244

[156] Tatham MH Geoffroy MC Shen L Plechanovova A Hattersley N Jaffray EG. Rnf4 is a poly-Sumo-Specific E3 ubiquitin ligase required for arsenic-induced pml degradation. Nat Cell Biol (2008) 10(5):538–46. doi: 10.1038/ncb1716 18408734

[157] Liu J Wu Z Han D Wei C Liang Y Jiang T. Mesencephalic astrocyte-derived neurotrophic factor inhibits liver cancer through small ubiquitin-related modifier (Sumo)Ylation-related suppression of nf-Kb/Snail signaling pathway and epithelial-mesenchymal transition. Hepatology (2020) 71(4):1262–78. doi: 10.1002/hep.30917 PMC7187412 31469428

[158] Kubota T Matsuoka M Chang TH Tailor P Sasaki T Tashiro M. Virus infection triggers sumoylation of Irf3 and Irf7, leading to the negative regulation of type I interferon gene expression. J Biol Chem (2008) 283(37):25660–70. doi: 10.1074/jbc.M804479200 PMC2533075 18635538

[159] Bentz GL Shackelford J Pagano JS. Epstein-Barr Virus latent membrane protein 1 regulates the function of interferon regulatory factor 7 by inducing its sumoylation. J Virol (2012) 86(22):12251–61. doi: 10.1128/jvi.01407-12 PMC3486478 22951831

[160] Chang TH Kubota T Matsuoka M Jones S Bradfute SB Bray M. Ebola Zaire Virus blocks type I interferon production by exploiting the host sumo modification machinery. PloS Pathog (2009) 5(6):e1000493. doi: 10.1371/journal.ppat.1000493 19557165 PMC2696038

[161] Izumiya Y Ellison TJ Yeh ET Jung JU Luciw PA Kung HJ. Kaposi's sarcoma-associated herpesvirus K-bzip represses gene transcription *Via* sumo modification. J Virol (2005) 79(15):9912–25. doi: 10.1128/jvi.79.15.9912-9925.2005 PMC1181544 16014952

[162] Izumiya Y Lin SF Ellison T Chen LY Izumiya C Luciw P. Kaposi's sarcoma-associated herpesvirus K-bzip is a coregulator of K-rta: Physical association and promoter-dependent transcriptional repression. J Virol (2003) 77(2):1441–51. doi: 10.1128/jvi.77.2.1441-1451.2003 PMC140808 12502859

[163] Izumiya Y Izumiya C Van Geelen A Wang DH Lam KS Luciw PA. Kaposi's sarcoma-associated herpesvirus-encoded protein kinase and its interaction with K-bzip. J Virol (2007) 81(3):1072–82. doi: 10.1128/jvi.01473-06 PMC1797516 17108053

[164] Izumiya Y Kobayashi K Kim KY Pochampalli M Izumiya C Shevchenko B. Kaposi's sarcoma-associated herpesvirus K-rta exhibits sumo-targeting ubiquitin ligase (Stubl) like activity and is essential for viral reactivation. PloS Pathog (2013) 9(8):e1003506. doi: 10.1371/journal.ppat.1003506 23990779 PMC3749962

[165] Chang PC Fitzgerald LD Van Geelen A Izumiya Y Ellison TJ Wang DH. Kruppel-associated box domain-associated protein-1 as a latency regulator for kaposi's sarcoma-associated herpesvirus and its modulation by the viral protein kinase. Cancer Res (2009) 69(14):5681–9. doi: 10.1158/0008-5472.Can-08-4570 PMC2731626 19584288

[166] Hagemeier SR Dickerson SJ Meng Q Yu X Mertz JE Kenney SC. Sumoylation of the Epstein-Barr virus Bzlf1 protein inhibits its transcriptional activity and is regulated by the virus-encoded protein kinase. J Virol (2010) 84(9):4383–94. doi: 10.1128/jvi.02369-09 PMC2863741 20181712

[167] Adamson AL Kenney S. Epstein-Barr Virus immediate-early protein Bzlf1 is sumo-1 modified and disrupts promyelocytic leukemia bodies. J Virol (2001) 75(5):2388–99. doi: 10.1128/jvi.75.5.2388-2399.2001 PMC114822 11160742

[168] Avey D Tepper S Pifer B Bahga A Williams H Gillen J. Discovery of a coregulatory interaction between kaposi's sarcoma-associated herpesvirus Orf45 and the viral protein kinase Orf36. J Virol (2016) 90(13):5953–64. doi: 10.1128/jvi.00516-16 PMC4907238 27099309

[169] Asai R Ohno T Kato A Kawaguchi Y. Identification of proteins directly phosphorylated by Ul13 protein kinase from herpes simplex virus 1. Microbes Infect (2007) 9(12-13):1434–8. doi: 10.1016/j.micinf.2007.07.008 17913541

[170] Purves FC Ogle WO Roizman B. Processing of the herpes simplex virus regulatory protein alpha 22 mediated by the Ul13 protein kinase determines the accumulation of a subset of alpha and gamma mrnas and proteins in infected cells. Proc Natl Acad Sci U.S.A. (1993) 90(14):6701–5. doi: 10.1073/pnas.90.14.6701 PMC47000 8393574

[171] Baek MC Krosky PM Pearson A Coen DM. Phosphorylation of the rna polymerase ii carboxyl-terminal domain in human cytomegalovirus-infected cells and in vitro by the viral Ul97 protein kinase. Virology (2004) 324(1):184–93. doi: 10.1016/j.virol.2004.03.015 15183065

[172] Long MC Leong V Schaffer PA Spencer CA Rice SA. Icp22 and the Ul13 protein kinase are both required for herpes simplex virus-induced modification of the Large subunit of rna polymerase ii. J Virol (1999) 73(7):5593–604. doi: 10.1128/jvi.73.7.5593-5604.1999 PMC112617 10364308

[173] Jenkins HL Spencer CA. Rna polymerase ii holoenzyme modifications accompany transcription reprogramming in herpes simplex virus type 1-infected cells. J Virol (2001) 75(20):9872–84. doi: 10.1128/jvi.75.20.9872-9884.2001 PMC114559 11559820

[174] Advani SJ Brandimarti R Weichselbaum RR Roizman B. The disappearance of cyclins a and b and the increase in activity of the G(2)/M-phase cellular kinase Cdc2 in herpes simplex virus 1-infected cells require expression of the Alpha22/U(S)1.5 and U(L)13 viral genes. J Virol (2000) 74(1):8–15. doi: 10.1128/JVI.74.1.8-15.2000 10590085 PMC111507

[175] Advani SJ Weichselbaum RR Roizman B. Cdc2 cyclin-dependent kinase binds and phosphorylates herpes simplex virus 1 U(L)42 DNA synthesis processivity factor. J Virol (2001) 75(21):10326–33. doi: 10.1128/jvi.75.21.10326-10333.2001 PMC114607 11581401

[176] Advani SJ Weichselbaum RR Roizman B. Herpes simplex virus 1 activates Cdc2 to recruit topoisomerase ii alpha for post-DNA synthesis expression of late genes. Proc Natl Acad Sci U.S.A. (2003) 100(8):4825–30. doi: 10.1073/pnas.0730735100 PMC153640 12665617

[177] Jacob RJ Roizman B. Anatomy of herpes simplex virus DNA viii. properties of the replicating DNA. J Virol (1977) 23(2):394–411. doi: 10.1128/jvi.23.2.394-411.1977 196115 PMC515842

[178] Fraser KA Rice SA. Herpes simplex virus immediate-early protein Icp22 triggers loss of serine 2-phosphorylated rna polymerase ii. J Virol (2007) 81(10):5091–101. doi: 10.1128/jvi.00184-07 PMC1900222 17344289

[179] Durand LO Advani SJ Poon AP Roizman B. The carboxyl-terminal domain of rna polymerase ii is phosphorylated by a complex containing Cdk9 and infected-cell protein 22 of herpes simplex virus 1. J Virol (2005) 79(11):6757–62. doi: 10.1128/jvi.79.11.6757-6762.2005 PMC1112163 15890914

[180] Morrison EE Wang YF Meredith DM. Phosphorylation of structural components promotes dissociation of the herpes simplex virus type 1 tegument. J Virol (1998) 72(9):7108–14. doi: 10.1128/jvi.72.9.7108-7114.1998 PMC109932 9696804

[181] Ren X Harms JS Splitter GA. Bovine herpesvirus 1 tegument protein Vp22 interacts with histones, and the carboxyl terminus of Vp22 is required for nuclear localization. J Virol (2001) 75(17):8251–8. doi: 10.1128/jvi.75.17.8251-8258.2001 PMC115069 11483770

[182] van Leeuwen H Okuwaki M Hong R Chakravarti D Nagata K O'Hare P. Herpes simplex virus type 1 tegument protein Vp22 interacts with taf-I proteins and inhibits nucleosome assembly but not regulation of histone acetylation by inhat. J Gen Virol (2003) 84(Pt 9):2501–10. doi: 10.1099/vir.0.19326-0 12917472

[183] Huang J You H Su C Li Y Chen S Zheng C. Herpes simplex virus 1 tegument protein Vp22 abrogates Cgas/Sting-mediated antiviral innate immunity. J Virol (2018) 92(15):e00841-18. doi: 10.1128/jvi.00841-18 29793952 PMC6052299

[184] Hamza MS Reyes RA Izumiya Y Wisdom R Kung HJ Luciw PA. Orf36 protein kinase of kaposi's sarcoma herpesvirus activates the c-jun n-terminal kinase signaling pathway. J Biol Chem (2004) 279(37):38325–30. doi: 10.1074/jbc.M400964200 15247271

[185] Lee CP Huang YH Lin SF Chang Y Chang YH Takada K. Epstein-Barr Virus Bglf4 kinase induces disassembly of the nuclear lamina to facilitate virion production. J Virol (2008) 82(23):11913–26. doi: 10.1128/jvi.01100-08 PMC2583647 18815303

[186] Cano-Monreal GL Wylie KM Cao F Tavis JE Morrison LA. Herpes simplex virus 2 Ul13 protein kinase disrupts nuclear lamins. Virology (2009) 392(1):137–47. doi: 10.1016/j.virol.2009.06.051 PMC2769575 19640559

[187] Kato A Yamamoto M Ohno T Tanaka M Sata T Nishiyama Y. Herpes simplex virus 1-encoded protein kinase Ul13 phosphorylates viral Us3 protein kinase and regulates nuclear localization of viral envelopment factors Ul34 and Ul31. J Virol (2006) 80(3):1476–86. doi: 10.1128/jvi.80.3.1476-1486.2006 PMC1346963 16415024

[188] Ng TI Ogle WO Roizman B. Ul13 protein kinase of herpes simplex virus 1 complexes with glycoprotein e and mediates the phosphorylation of the viral fc receptor: Glycoproteins e and I. Virology (1998) 241(1):37–48. doi: 10.1006/viro.1997.8963 9454715

[189] Van Cleemput J Koyuncu OO Laval K Engel EA Enquist LW. Crispr/Cas9-constructed pseudorabies virus mutants reveal the importance of Ul13 in alphaherpesvirus escape from genome silencing. J Virol (2021) 95(6):e02286-20. doi: 10.1128/jvi.02286-20 33361431 PMC8094956

[190] Lukac DM Renne R Kirshner JR Ganem D. Reactivation of kaposi's sarcoma-associated herpesvirus infection from latency by expression of the orf 50 transactivator, a homolog of the ebv r protein. Virology (1998) 252(2):304–12. doi: 10.1006/viro.1998.9486 9878608

[191] Guito J Lukac DM. Kshv rta promoter specification and viral reactivation. Front Microbiol (2012) 3:30. doi: 10.3389/fmicb.2012.00030 22347875 PMC3278982

[192] Damania B Jeong JH Bowser BS DeWire SM Staudt MR Dittmer DP. Comparison of the Rta/Orf50 transactivator proteins of gamma-2-Herpesviruses. J Virol (2004) 78(10):5491–9. doi: 10.1128/jvi.78.10.5491-5499.2004 PMC400334 15113928

[193] Simpson S Fiches G Jean MJ Dieringer M McGuinness J John SP. Inhibition of Tip60 reduces lytic and latent gene expression of kaposi's sarcoma-associated herpes virus (Kshv) and proliferation of kshv-infected tumor cells. Front Microbiol (2018) 9:788. doi: 10.3389/fmicb.2018.00788 29740418 PMC5928232

[194] Kedes DH Lagunoff M Renne R Ganem D. Identification of the gene encoding the major latency-associated nuclear antigen of the kaposi's sarcoma-associated herpesvirus. J Clin Invest (1997) 100(10):2606–10. doi: 10.1172/jci119804 PMC508462 9366576

[195] Shamay M Liu J Li R Liao G Shen L Greenway M. A protein array screen for kaposi's sarcoma-associated herpesvirus Lana interactors links Lana to Tip60, Pp2a activity, and telomere shortening. J Virol (2012) 86(9):5179–91. doi: 10.1128/jvi.00169-12 PMC3347335 22379092

[196] Sylvester PA Jondle CN Stoltz KP Lanham J Dittel BN Tarakanova VL. Conserved gammaherpesvirus protein kinase counters the antiviral effects of myeloid cell-specific Stat1 expression to promote the establishment of splenic b cell latency. J Virol (2021) 95(17):e0085921. doi: 10.1128/jvi.00859-21 34132573 PMC8354328

[197] Liu T Khanna KM Chen X Fink DJ Hendricks RL. Cd8(+) T cells can block herpes simplex virus type 1 (Hsv-1) reactivation from latency in sensory neurons. J Exp Med (2000) 191(9):1459–66. doi: 10.1084/jem.191.9.1459 PMC2213436 10790421

[198] Doherty PC Christensen JP Belz GT Stevenson PG Sangster MY. Dissecting the host response to a gamma-herpesvirus. Philos Trans R Soc Lond B Biol Sci (2001) 356(1408):581–93. doi: 10.1098/rstb.2000.0786 PMC1088446 11313013

[199] Hollingworth R Horniblow RD Forrest C Stewart GS Grand RJ. Localization of double-strand break repair proteins to viral replication compartments following lytic reactivation of kaposi's sarcoma-associated herpesvirus. J Virol (2017) 91(22):e00930-17. doi: 10.1128/jvi.00930-17 28855246 PMC5660498

[200] Zimmermann A Wilts H Lenhardt M Hahn M Mertens T. Indolocarbazoles exhibit strong antiviral activity against human cytomegalovirus and are potent inhibitors of the Pul97 protein kinase. Antiviral Res (2000) 48(1):49–60. doi: 10.1016/s0166-3542(00)00118-2 11080540

[201] Gershburg E Hong K Pagano JS. Effects of maribavir and selected indolocarbazoles on Epstein-Barr virus protein kinase Bglf4 and on viral lytic replication. Antimicrob Agents Chemother (2004) 48(5):1900–3. doi: 10.1128/aac.48.5.1900-1903.2004 PMC400567 15105156

[202] Goswami R Gershburg S Satorius A Gershburg E. Protein kinase inhibitors that inhibit induction of lytic program and replication of Epstein-Barr virus. Antiviral Res (2012) 96(3):296–304. doi: 10.1016/j.antiviral.2012.09.021 23058855 PMC3513622

[203] Hoke GD Draper K Freier SM Gonzalez C Driver VB Zounes MC. Effects of phosphorothioate capping on antisense oligonucleotide stability, hybridization and antiviral efficacy versus herpes simplex virus infection. Nucleic Acids Res (1991) 19(20):5743–8. doi: 10.1093/nar/19.20.5743 PMC328985 1658742

[204] Crooke RM Hoke GD Shoemaker JE. *In vitro* toxicological evaluation of Isis 1082, a phosphorothioate oligonucleotide inhibitor of herpes simplex virus. Antimicrob Agents Chemother (1992) 36(3):527–32. doi: 10.1128/aac.36.3.527 PMC190551 1377898

[205] Sun X Bristol JA Iwahori S Hagemeier SR Meng Q Barlow EA. Hsp90 inhibitor 17-dmag decreases expression of conserved herpesvirus protein kinases and reduces virus production in Epstein-Barr virus-infected cells. J Virol (2013) 87(18):10126–38. doi: 10.1128/jvi.01671-13 PMC3754017 23843639

[206] Lv L Liu X Jiang C Wang X Cao M Bai J. Pathogenicity and immunogenicity of a Gi/Ge/Tk/Ul13-Gene-Deleted variant pseudorabies virus strain in swine. Vet Microbiol (2021) 258:109104. doi: 10.1016/j.vetmic.2021.109104 34004569

[207] Olotu FA Soliman MES. Immunoinformatics prediction of potential b-cell and T-cell epitopes as effective vaccine candidates for eliciting immunogenic responses against Epstein-Barr virus. BioMed J (2021) 44(3):317–37. doi: 10.1016/j.bj.2020.01.002 PMC8358216 34154948

[208] Uldrick TS Polizzotto MN Aleman K O'Mahony D Wyvill KM Wang V. High-dose zidovudine plus valganciclovir for kaposi sarcoma herpesvirus-associated multicentric castleman disease: A pilot study of virus-activated cytotoxic therapy. Blood (2011) 117(26):6977–86. doi: 10.1182/blood-2010-11-317610 PMC3143547 21487108

[209] Wang QJ Jenkins FJ Jacobson LP Kingsley LA Day RD Zhang ZW. Primary human herpesvirus 8 infection generates a broadly specific Cd8(+) T-cell response to viral lytic cycle proteins. Blood (2001) 97(8):2366–73. doi: 10.1182/blood.v97.8.2366 11290599

[210] Robey RC Lagos D Gratrix F Henderson S Matthews NC Vart RJ. The Cd8 and Cd4 T-cell response against kaposi's sarcoma-associated herpesvirus is skewed towards early and late lytic antigens. PloS One (2009) 4(6):e5890. doi: 10.1371/journal.pone.0005890 19536280 PMC2691989

